# Mouse models characterize *GNAO1* encephalopathy as a neurodevelopmental disorder leading to motor anomalies: from a severe G203R to a milder C215Y mutation

**DOI:** 10.1186/s40478-022-01312-z

**Published:** 2022-01-28

**Authors:** Denis Silachev, Alexey Koval, Mikhail Savitsky, Guru Padmasola, Charles Quairiaux, Fabrizio Thorel, Vladimir L. Katanaev

**Affiliations:** 1 A.N. Belozersky Research Institute of Physico-Chemical Biology, Moscow State University, 119992 Moscow, Russia; 2 V.I. Kulakov National Medical Research Center of Obstetrics, Gynecology and Perinatology, Moscow, 117997 Russia; 3 Department of Cell Physiology and Metabolism, Faculty of Medicine, Translational Research Center in Oncohaematology, University of Geneva, 1211 Geneva, Switzerland; 4 School of Biomedicine, Far Eastern Federal University, 690090 Vladivostok, Russia; 5 Department of Basic Neuroscience, Faculty of Medicine, University of Geneva, 1211 Geneva, Switzerland; 6 Transgenesis Core Facility, Faculty of Medicine, University of Geneva, 1211 Geneva, Switzerland

**Keywords:** *GNAO1*, Gαo, Encephalopathy, Mouse model, Dominant, Epilepsy, Hyperkinetic movement disorder, Neurodevelopmental, Neuronal precursors

## Abstract

*GNAO1* encephalopathy characterized by a wide spectrum of neurological deficiencies in pediatric patients originates from de novo heterozygous mutations in the gene encoding Gαo, the major neuronal G protein. Efficient treatments and even the proper understanding of the underlying etiology are currently lacking for this dominant disease. Adequate animal models of *GNAO1* encephalopathy are urgently needed. Here we describe establishment and characterization of mouse models of the disease based on two point mutations in *GNAO1* with different clinical manifestations. One of them is G203R leading to the early-onset epileptic seizures, motor dysfunction, developmental delay and intellectual disability. The other is C215Y producing much milder clinical outcomes, mostly-late-onset hyperkinetic movement disorder. The resultant mouse models show distinct phenotypes: severe neonatal lethality in *GNAO1[G203R]/* + mice *vs.* normal vitality in *GNAO1[C215Y]/* + . The latter model further revealed strong hyperactivity and hyperlocomotion in a panel of behavioral assays, without signs of epilepsy, recapitulating the patients’ manifestations. Importantly, despite these differences the two models similarly revealed prenatal brain developmental anomalies, such as enlarged lateral ventricles and decreased numbers of neuronal precursor cells in the cortex. Thus, our work unveils *GNAO1* encephalopathy as to a large extent neurodevelopmental malady. We expect that this understanding and the tools we established will be instrumental for future therapeutic developments.

**Supplementary Information:**

The online version contains supplementary material available at 10.1186/s40478-022-01312-z.

## Introduction

*GNAO1* encephalopathy is a group of neurological disorders, manifesting in infants and children and caused by heterozygous and mainly de novo mutations in *GNAO1*-the gene encoding the major neuronal G protein Gαo [1–4]. One of the hotspots of mutations, c.607G > A producing the Gαo[G203R] protein variant, leads to the most severe manifestations of the disease: early onset (sometimes days after birth) epileptic seizures, severe motor dysfunctions, developmental and intellectual delay, and brain malformations (Table 1). This mutation has been clinically described in 7 patients [1, 2, 5–7] (Table 1); a recent survey reported 12 patients with the Gαo[G203R] substitution [3].

**Table 1:** *C215Y* and *G203R* patients described in the literature. Patient 2 is the mother of patient 3. All other cases are de novo mutations

Pat. #	Sex	Nucleotide change	Amino acid change	Age of onset (age at publication)	Epilepsy	Movement disorder	Intellectual disability / developmental delay	Brain alterations (MRI)	Reference
1	M	c.644G > A	C215Y	12y (42y)	No	Myoclonus dystonia	No	No	[9]
2	F	c.644G > A	C215Y	5y (66y)	No	Non-progressive generalized dystonia, myoclonus, pyramidal syndrome	No	NA	[8]
3	M	c.644G > A	C215Y	3y (31y)	No	Non-progressive generalized dystonia, myoclonus	Mild intellectual disability	No	[8]
4	F	c.607G > A	G203R	7 m (8y)	Yes	Severe chorea, athetosis	Developmental delay	Delayed myelination, thin corpus calossum	[2]
5	F	c.607G > A	G203R	7d (14 m)	Yes	Severe chorea	Developmental delay	Cerebral atrophy, delayed myelination	[5]
6	M	c.607G > A	G203R	1 m (5y)	yes	Severe chorea, athetosis	Developmental delay	Progressive atrophy	[6]
7	F	c.607G > A	G203R	3 m (6y)	Yes	Dyskinesia, dystonia	Motor developmental delay, intellectual disability	Progressive atrophy, thin corpus calossum	[7]
8	F	c.607G > A	G203R	9d (4y)	Yes	Dyskinesia, dystonia	Motor developmental delay, intellectual disability	Mild atrophy	[7]
9	F	c.607G > A	G203R	1d (3y)	Yes	Generalized hypotonia, mild dyskinesia, generalized dystonia, chorea	Severe neurodevelopmental delay	Thin corpus calossum	[1]
10	M	c.607G > A	G203R	12d (5y)	Yes	Hypotonia, dyskinesia, hyperkinesia, status dystonicus, dystonia	Developmental delay	Hypomyelination, brain atrophy	[1]

NA – data not available

On the other extreme of the reported *GNAO1* mutations, c.644G > A resulting in the Gαo[C215Y] protein variant has a relatively late age of onset (3 to 12 years), no signs of epilepsy, mild or no intellectual disability. Clinically, the C215Y mutation manifests as various types of motor dysfunction, manly myoclonus dystonia, which, however, permit the patients to work and have families [8, 9] (Table 1).

No curative but only symptomatic treatments with mixed, mostly mild and temporary, treatment outcomes have so far been available to *GNAO1* patients [3, 10, 11]. Development of curative therapies has as prerequisites the understanding of the molecular etiology of the disease, as well as the establishment of proper animal models. We thus set to model these two *GNAO1* mutations-the severe G203R and the mild C215Y-in the mouse. Our results reveal the early postnatal lethality of *GNAO1[G203R]/* + but not *GNAO1[C215Y]/* + mice, varying brain developmental malformations in both genotypes, and hyperlocomotion behavior of the *C215Y/* + mutant mice. This latter phenotype can be viewed as the mouse analog of the hyperkinetic movement disorder of the human C215Y patients [8, 9]. Our work thus describes important rodent models of *GNAO1* encephalopathy and further highlights it as to a large extent neurodevelopmental disease. These models will be instrumental in the drug discovery/development targeting *GNAO1* encephalopathies.

## Materials and methods

### Animal experimentation

All experiments involving animals were conducted in accordance with the Swiss law and authorized by the Swiss Federal Food Safety and Veterinary Office (authorizations #GE/17/20 and #GE/25/20).

### CRISPR/Cas9 mutagenesis

To generate *GNAO1[G203R]* (c.607G > A) and *GNAO1[C215Y]* (c.644G > A) mutant mice, we reconstituted CRISPR ribonucleoprotein complexes (RNP) containing the Cas9 protein with following gRNAs: gRNA_G203R: 5ʹ-TGCAGGCTGTTTGACGTCGG-3ʹ; gRNA_C215Y: 5ʹ-CCGTGACATCCTCAAAGCAG-3ʹ. As donor template, we used the following ssDNAs: ssDNA(G203R): 5ʹ-ATGGCCGTGACATCCTCAAAGCAGTGGATCCACTTCTTGCGTTCAGATCGCTGGCCgCgGACGTCAAACAG CCTGCAGGGAGTCAGGGAAAGCTGT-3ʹ; ssDNA(C215Y): 5ʹ-GGCCAGCGATCTGAACGCAAGAAGTGGATCCACTaCTTTGAGGATGTCACGGCCATCATCTTCTGTGTCGCAC TCAGCGGCTATGACCAGG-3ʹ. Cas9, gRNA and ssDNA were from Integrate DNA Technologies (Zurich, Switzerland). RNP complexes were co-injected with the appropriate donor ssDNA in C57BL/6 J one cell embryos before transfer into CD1 foster mothers. In attempts to establish the *GNAO1[G203R]* line, we performed three independent injection sessions resulting in a total of 377 transferred embryos. In total, 57 F0 pups were found in the cages after birth, either as living or dead animals, among which majority had *G203R/* + , *G203R/G203R*, or *G203R/[frame shift truncation]* genotypes. In total, nine F0 *G203R/* +  were born, of which seven were found still alive the night after birth, but five of them perished. *G203R* E18.5 embryos for the histological analysis were obtained through a transgenesis procedure described above, but followed by caesarian section and transcardial fixation of the embryos. The lethality associated with the G203R mutation cannot be due to off-target effects for the following two reasons. First, considering that the off-target events are sporadic [12] and assuming their 50% frequency [13] we arrive at the zero probability of a reiterating secondary lethal mutation to be observed in the 16 *G203R/* + or *G203R/G203R* pups. Second, excluding a non-sporadic off-target mutations, the fact that only *G203R/* + and *G203R/G203R* F0 pups were neonatal lethal, as opposed to their littermates either wt/wt or containing a wt and a truncated allele of *GNAO1*, argues for the specific lethality caused by the G203R mutation, rather than potential off-target effects of gRNAs.

To generate the *C215Y* line, 113 injected embryos were transferred in 1 session that resulted in the total of 30 F0 pups, among which we obtained one *C215Y/* + and six *C215Y/C215Y* animals.

### Mouse genotyping

Genomic DNA was isolated from fingers, pieces of tails, ears or legs of mice of different genotypes as described previously [14]. Induced point mutations and indels were verified by sequencing of PCR fragments obtained with the primers Fwd1_mGNAO1 (GACAGGTGTCACAGGGGATG) and Rev2_mGNAO1 (GGGCAGACAAGTGAACAAGTGAA). Sequencing was performed with the primers Rev1_mGNAO1 (TCCTAGCCAAGACCCCAACT) or Fwd2_mGNAO1 (TCATCTGTCAGCCTGTTCCTCAC). All PCRs were performed using Phusion High-Fidelity DNA Polymerase (New England Biolabs, Ipswich MA, USA) following the manufacturer's instructions.

Total RNA was isolated with the NucleoSpin RNA kit (Macherey–Nagel, Dueren, Germany) from pieces of brains or pieces of bodies. cDNA was synthesized by priming with oligo-dT with RevertAid Reverse Transcriptase (Thermo Fisher Scientific, Waltham MA, USA, cat. #EP0441) following the manufacturer’s instructions.

160 bp fragments partially overlapping 5th and 6th exons of GNAO1 were amplified from cDNA with primers mGNAO1_comfw (ACATCCTCCGAACCAGAGTCA) and mGNAO1_comrev (GTGCGACACAGAAGATGATGG) and then sequenced.

### Fixation by transcardial perfusion

The animals were anaesthetized by isoflurane induction dose (5%) for 5 min followed by a maintenance dose of 2%. The C-section was performed on pregnant mice, the pups were taken, analgezied (carprofen IP, 80–100 µl of 0.5 mg/ml solution, 5 mg/kg) and immediately anesthetized by 5 min isoflurane induction dose (3%) in the chamber. Afterwards, they were maintained at 2%. After at least 10 min since carprofen injection, the pups were removed from chamber one by one, placed under the individual isoflurane mask and had an intracardiac perfusion of 1xPBS followed by fixation of the tissue with 4% paraformaldehyde in 1xPBS.

### Histological analysis and immunohistochemistry

Brains from fixed E18.5 embryos were collected, fixed in 4% PFA, paraffin embedded and cut into 5 μm thick sections. The sections were mounted on glass slides and stored at 4 °C. Sections were deparaffinized, rehydrated and stained by hematoxylin–eosin or cresyl violet. For immunohistochemistry, sagittal sections were prepared from + / + (n = 4), C215Y/ + (n = 4), C215Y/C215Y (n = 5), G203R/ + (n = 1) and G203R/G203R (n = 2) samples. For the staining, the slides were first deparaffinized in three changes each of xylene, sequence of water mixtures of EtOH with decreasing concentrations (95%, 70% and 50%) and finally water. Subsequently, antigen retrieval was performed in 20 mM Tris–EDTA, pH 9.0 with 0.1% Tween-20 by heating up the slides to 95 °C for 20 min and gradual cooling to the room temperature. Further, the slides were blocked in 1xPBS/ 0.1% Triton X-100 with 1% of normal horse serum for 30 min at room temperature. They were then incubated with the different primary antibodies: Tbr1 (1:100, Abcam #ab31940); Ctip2 (1:500, Abcam #ab18465); Brn2 (1:200, Santa Cruz Biotechnologies #sc-393324); Tbr2 (1:100, Abcam #ab23345), Reelin (1:1000, Abcam # ab230820), Nestin (1:200, Abcam # ab6142), Cleaved Caspase-3 (1:200, Cell signaling #9661). Secondary antibodies were conjugates of Alexa Fluor 488, Alexa Fluor 594, and Alexa Fluor 647 (1:250). DAPI (4’,6’-diamidino-2-phenylindole) was used as nuclear counterstaining at 1 µg/ml. Finally, slices were washed and mounted in Fluorescent Mounting Medium (Dako Cytomation). Genotype for samples was checked at the end of the experimental process. Cell counting was performed on rostral cortical regions (at the level of the striatum). At least three serial sections from three different animals for each genotype were photographed using an LSM710 confocal microscope or an Axio-Scan.Z1 slide scanner (Carl Zeiss). The number of relevant cells was calculated using the deep-learning algorithm Cellpose, which allows automatically counting cells in the cortex [15], or manually using the ImageJ. Nestin immunoreactivity was measured in each ROI as average fluorescence intensity.

Hematoxylin and eosin images analysis was carried out using NIH ImageJ software (Wayne Rasband, NINDS, NIH). The following parameters were measured: area of brain slices; cortex thickness (perpendicular to the lateral ventricular region) and area of the lateral ventricles.

### Behavioral Assessment

The study was conducted on mice of both sexes with genotypes C215Y/ + (n = 17), C215Y/C215Y (n = 6) and their + / + (n = 8) littermates at the age of 8–12 weeks. Mice were left for 30 min prior to the experiment in the room to allow acclimatization.

#### Open field test

Mice were placed in a square open field (42 × 42 cm) and were allowed to freely explore the open field for a 30-min period in 11-lx illumination condition. Total distance traveled, velocity and spent time in center of arena during the session were automatically analyzed (Ethovision, Noldus). The arena was cleaned with 70% ethanol and dried between each test.

#### Elevated plus maze

The elevated plus maze consisted of a platform with four opposite arms (40 cm), two of them open and two closed (enclosed by 15 cm high walls). The apparatus was elevated at 55 cm from the floor. The task was recorded and analyzed with the software Ethovision (Noldus) and we measured the time spent in each arm in trials of 10 min. The luminosity of the room was 11 lx in the open arms.

#### Swimming tank test

To measure swimming behavior, we used a swimming tank apparatus as previously described [16]. The tank was filled with water (26–27 °C) until the escape platform remained elevated 1-2 cm above the water level. At the opposite side of the tank, a vertical red line indicated the starting point located at 60 cm from the platform. The task consisted of three consecutive trials (~ 10 s between trails), performed daily from day 1 to 3. We measured the time to swim the 60 cm of distance from the red line to the platform. The task was recorded and analyzed with the software VLC Media Player.

#### Rotarod test

To assess motor skills, we used the rotarod apparatus (Ugo Basile, Biological Research Apparatus) consisted of a plastic roller with small grooves running along its turning axis. Mice were subjected to two trials per day for four consecutive days with 20 min intervals between trials. The protocol included a classical accelerated rotarod [17] sequence ramping up from five rotations per minute (RPM) to 40 RPM within 240 s for a maximum duration of 300 s. We scored the mouse latency to fall in seconds of each last trial session per day. Mice that did not fall during experiment were assigned the time of 300 s.

#### Pole test

The pole test was performed as previously described [18] with minor modifications. A 50 cm pole with a diameter of 10 mm was placed in animal cage. The mouse was put head-upward on the top of a vertical rough-surfaced pole; the time to descend to the floor and turn time at the end of the pole were measured. Experimental session consisted of 5 trials with 15 s interval trial break.

### Analysis of the *C215Y/* + behavior by motion sequencing (MoSeq) algorithm

The original Moseq script [19, 20] was obtained from Michael Schartner [21]. The MATLAB kinect recording script and MoSeq processing modules were modified to ensure recording at 30fps over the entire 20 min recording session and compatibility with Kinect 2 data. To ensure fps stability, the data over 20 min was acquired in ~ 20 s periods followed by 1-2 s interruptions necessary for storage. Additionally, the manual preprocessing control step was added to allow monitoring and removal of several remaining mouse tracking and recognition artifacts. Overall, ten *C215Y/* + , five *C215Y/C215Y* and six wild-type (wt) 2 months-old littermates were analyzed this way. PCA fit of the entire merged dataset was performed with 15 dimensions and subsequent state analysis by ARHMM with 15 states revealed that assignment of the states for the data set of this size had partially stochastic character. The fit was culminating at stable “equilibrium”, unchanged by increased iteration number, but for each independent run this resulted in somewhat different assignment of the states for certain frames. This stochastic assignment was independent of PCA fit and states number in ARHMM model. Thus, in order to avoid bias resulting from choosing a single run to interpret and better delineate the syllable borders, we have performed 8 independent ARHMM model fits and thus obtained 8 independent values of states per frame as its unique syllable signature. > 50,000 of such unique signatures were clustered via Scipy hierarchy clustering using WPGMA algorithm and manually split into 44 clusters obtaining final syllables. For these syllables we have subsequently evaluated frequency as a number of times it is called per minute of mouse activity, mean length of uninterrupted assignment in seconds and a total proportion of this syllable over the entire recorded time.

### Large-scale high-density electroencephalography (EEG)

Surface epicranial EEGs were recorded in head-fixed, awake animals with a high-density grid of 32 stainless steel electrodes (see Additional file 1: Fig. S11A) covering the entire skull surface as described previously [22]. Briefly, a head-post was first placed under anesthesia (Medetomidin 5 mg/kg body weight (bw), Midazolam 5 mg/kg bw, Fentanyl 0.05 mg/kg bw) in order to allow head-fixation and the placement of the epicranial grid during the subsequent EEG recordings. Recording sessions then took place after a period of 4d of head-fixation training to allow acclimatization of the animals to the experimental setup. Electrophysiological differential recordings were acquired during sessions of 1 h with a Digital Lynx SX (Neuralynx, USA) at a sampling rate of 4 kHz and with a 2 kHz low-pass. The ground electrode was placed above the nasal bone and the reference electrode was placed on the midline between parietal bones and all signals were then calculated against the average reference offline. EEG signals were then scrutinized by an experimenter blind to the animal condition (wt or mutant mouse) to detect the presence of paroxysmal discharges reminiscent of ictal or interictal-like activities using similar analyses tools as previously described previously [22]. In addition to the reviewer visual detection of seizure like patterns and epileptic spikes, we applied an automatic detection of fast ripples, a biomarker of epileptogenic brain regions [23, 24]. This detector identifies events with 4 consecutive oscillations 3 times higher in amplitude than the SD of the 250 ms surrounding baseline after filtering the EEG signals between 200 and 550 Hz using an order 2 Butterworth filter. All identified events were then visually confirmed in the filtered and unfiltered data as described in previous publications with the kainate mouse model [22].

### Pentylenetetrazol (PTZ)-induced convulsion test

We used a PTZ kindling model of epilepsy to achieve a sub-convulsionary state [25]. Briefly, *C215Y/* + and + / + mice were injected with two doses of 30 and 35 mg/kg every other day of PTZ (Sigma, P6500) which were enough to achieve a sub-convulsionary state for both genotypes. The mice were scored over a period of 30 min after PTZ injection according to the intensity of the behavioral seizure as described [26].

## Results

### G203R mutation in the *GNAO1* gene demonstrates early postnatal lethality

**Fig. 1:**
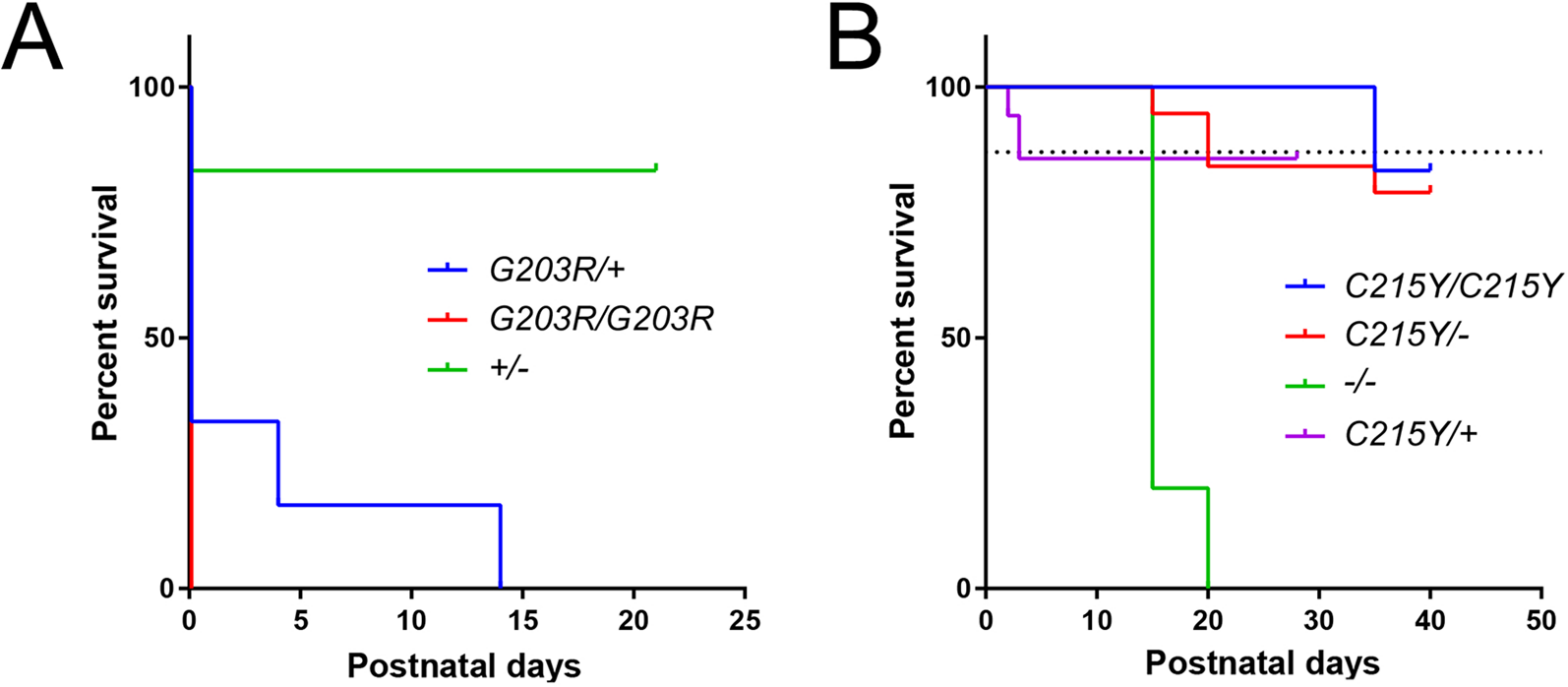
Survival rate of F0 mice of different genotypes after transgenesis. **A** *G203R* F0 mice, either homozygous or heterozygous, reveal dramatic postnatal lethality. Only two *G203R*/ + mice lived past postnatal day 1. **B** In contrast, heterozygous or even homozygous F0 *G215Y* mice revealed the survival not different from fully wt mice (dotted line). *GNAO1*^*−/−*^ loss-of-function animals generated as a by-product of the transgenesis showed reduced survival. “−” designates a truncated allele resulting from CRISPR-mediated indels

We first attempted to create G203R (c.607G > A)-bearing mutant mice using the CRISPR/Cas9-based technique (see Materials and Methods). We performed 3 injection sessions of the CRISPR-Cas9 reagents and template designed to introduce target mutation in the mouse *GNAO1* gene. The results are summarized in the survival graph (Fig. 1A). Overall, we obtained 9 homozygous (*G203R/G203R*) and 7 heterozygous (*G203R/* +) F0 pups. All of the homozygous pups were dead within one day after birth, whereas 2 out of 7 heterozygous pups survived until P4 and P14 and were found spontaneously dead without signs of external damage. Of the littermates, the pups surviving until weaning were either + / + (wt/wt) or contained a wild-type (wt) and a truncated allele of *GNAO1* resulting from CRISPR-mediated indels.

In parallel, we attempted to similarly introduce the C215Y (c.644G > A) point mutation. The resultant F0 mice and progeny were viable and fertile, both as heterozygotes and as homozygotes (Fig. 1B). As a by-product of the trangenesis, *GNAO1*^−/−^ loss-of-function (homozygous for indel mutations leading to truncated unfunctional protein) animals were generated, showing a reduced survival (Fig. 1B) in accordance with earlier reports [27]. The normal viability and fertility of the mutant C215Y *GNAO1* mice permitted us to establish a stable colony and to perform a set of behavioral experiments, detailed below.

Additional file 1: Fig. S1 shows the proper expression of the mutant (*G203R* and *C215Y*) alleles, alongside with the wt allele, in the mutant animals. Note that the peak height of the mutant nucleotide is nearly identical to that of wt in both mutants, speaking of near-equal expression of both alleles. Taken together, these findings prove that the G203R mutation-unlike the C215Y mutation–even in heterozygous state is postnatally lethal.

### C215Y mutation leads to altered brain morphology in E18.5 embryos

**Fig. 2:**
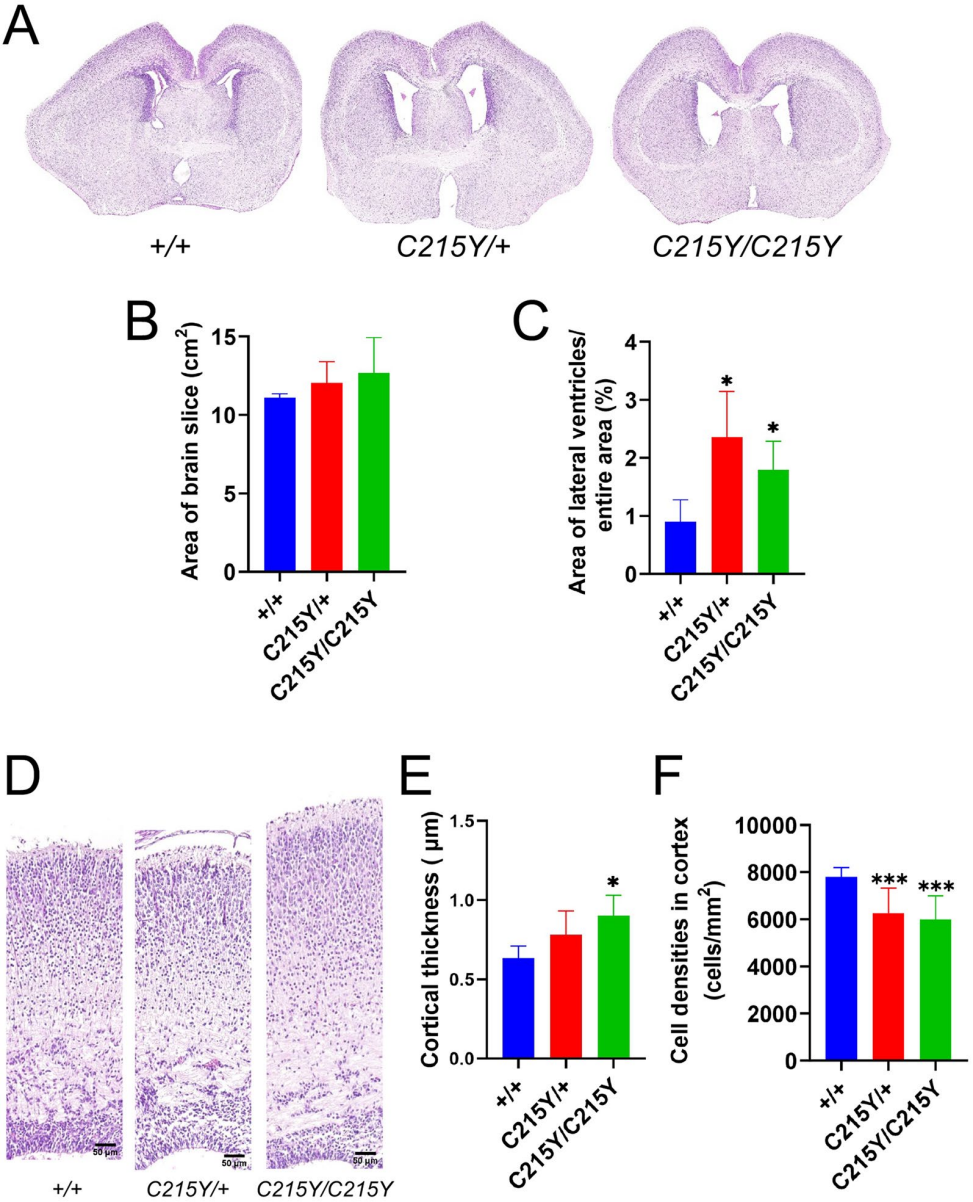
Effects of homo- and heterozygous *C215Y* mutation on brain morphology. **A**–**C** Hematoxylin and eosin staining of coronal brain sections from *C215Y/* + , *C215Y/C215Y*, and wt (+ / +) littermates at the embryonic day 18.5 (**A**) reveals insignificant changes in the overall brain area (**B**) but enlarged lateral ventricles in the mutants (**C**). **D**–**F** Coronal sections through the motor cortex (**D**) show differences in the cortical thickness between wt and *C215Y/C215Y* mice (**E**). Quantification of the cell density in this region reveals that it is decreased in the two mutant genotypes (**F**). Data are presented as mean ± SD, n = 3–5 animals. *p < 0.05, **p < 0.01, ***p < 0.005, significance from the wt is assessed by t-test. Scale bar: 50 µm

Abnormal structure and function of the cerebral cortex have been reported across studies of patients with *GNAO1* mutations [1, 2, 5–7]. Thus, in the newly created disease model based on the C215Y mutation we first analyzed the brain morphology. Considering that the G203R mutation is lethal in the early postnatal stage, we chose to analyze embryos at the late gestation age of 18.5 days to allow eventual comparison between the two models (see below) while approaching the brain development level of new-born humans. We analyzed the coronal brain sections of heterozygous (*C215Y/* +), homozygous (*C215Y/C215Y*) and wt littermates at 18.5 days of gestation stained with H&E (Fig. 2A), and quantified the areas of the brain slices and the size of lateral ventricles. No significant difference in the area of the coronal section of the brain was found across the genotypes (Fig. 2B). The lateral ventricles were significantly enlarged in the *C215Y/* + and *C215Y/C215Y* mice compared with those of the wt littermates (Fig. 2C). Subsequent analysis revealed differences in the motor cortex thickness between *C215Y/C215Y* and wt littermates (Fig. 2D,E). To evaluate whether the cell numbers were changed in the entire cortex, the total number of cortical cells was quantified by the deep learning-based segmentation algorithm Cellpose [15] in brain slices stained with DAPI, revealing a significant decrease in the cell densities in the two mutant genotypes (Fig. 2F) while keeping the cell numbers unchanged (ca. 12,000 cells for each genotype).

In opposite to the changes in the cortex induced by the *C215Y* mutation, we did not find aberrations in two other brain regions analyzed: striatum (Additional file 1: Fig. S2) and hippocampus (Additional file 1: Fig. S3).

### Reduced numbers of cortical progenitor cells underlie brain morphology aberrations in *C215Y* mutants

Complete knockout of *GNAO1* impaired the cerebellar cortical development in mice [28] asserting key role of Gαo in brain compartment development. We thus investigated the potential influence of the C215Y mutation in brain development, particularly in the context of the cortex. Changes in the cellular composition in the cortex of mice bearing the C215Y mutation were analyzed quantitatively looking at different cell markers in each layer of the cortex at the stage E18.5 [29]. Projection neurons residing in deeper layers of the cortex were stained by Tbr1 (layer VI) and Ctip2 (layer V); upper layers were analyzed using Brn2 (present in layers V to II but mostly present in the layers II/III) and Reelin for identification of Cajal-Retzius cells lying in the most superficial molecular layer [30, 31].

**Fig. 3:**
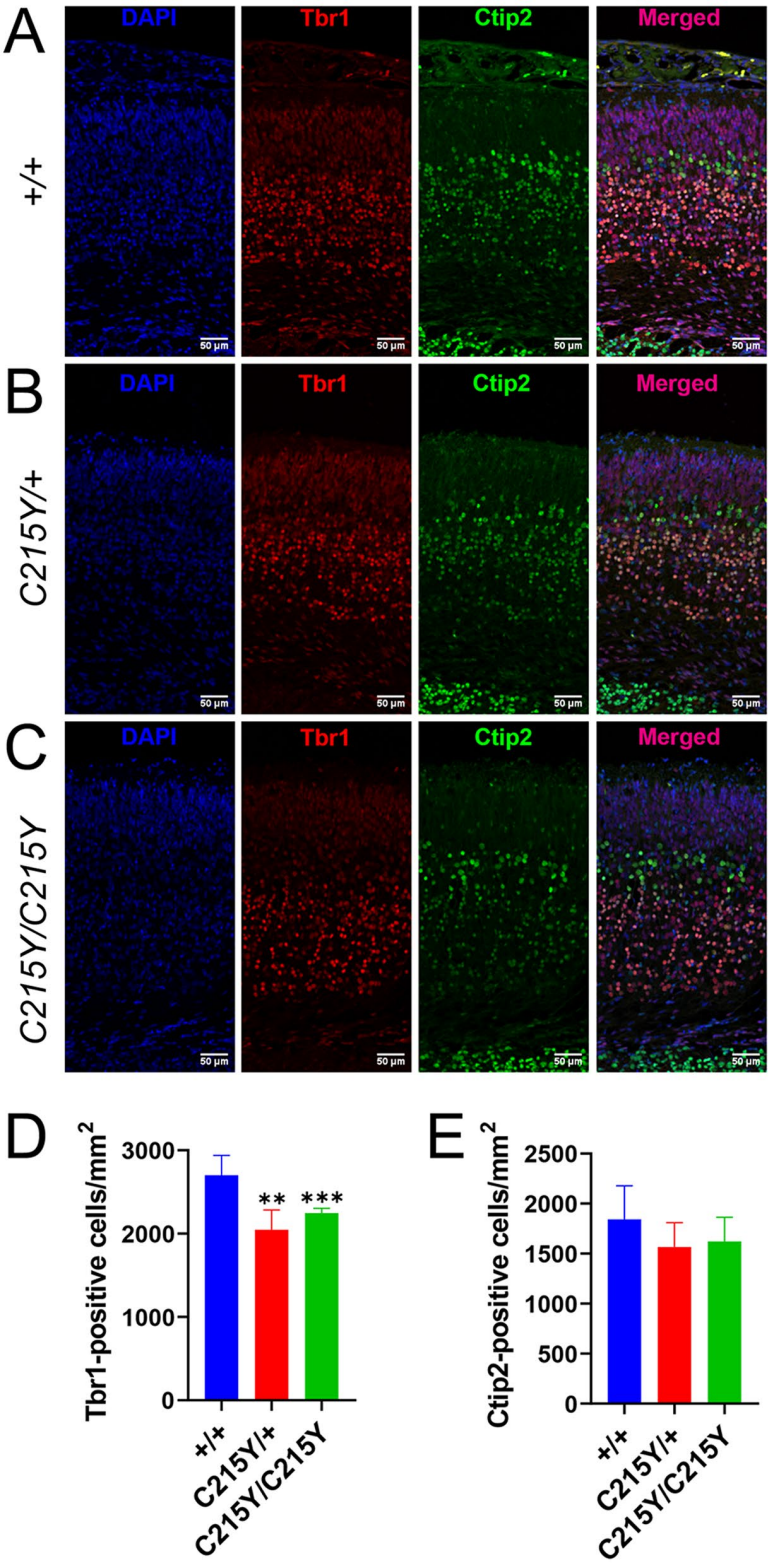
*C215Y* mice show decreased number of neurons in deep layers of the cortex. **A**–**C** Staining of motor cortex brain sections from E18.5 wt (+ / + , **A**), *C215Y/* + (**B**), and *C215Y/C215Y* (**C**) mice for DAPI (blue), Tbr1 (layer VI, red), and Ctip2 (weak in layer VI, strong in layer V, green). **D** Quantification of Tbr1-positive cells’ density. **E** Quantification of Ctip2-positive cells’ density. Data are presented as mean ± SD; n = 3–5 animals; **p < 0.01, ***p < 0.005, significance from the wt is assessed by t-test. Scale bar: 50 µm

We found that the layer VI (Tbr1-positive), the deepest and the first to be generated during brain development, had significantly lesser cell density in both homozygous and heterozygous *C215Y* mice compared to their wt littermates (Fig. 3A–D). In contrast, we found no statistically significant difference in the number of Ctip2-positive cells in the layer V (Fig. 3A–C,E). A decrease in the number of Brn2-positive cells in layers II–V was seen in the mutant genotypes, becoming statistically significant for the homozygous mutants (Additional file 1: Fig. S4A, C). In the molecular layer of the cortex, there was a similar trend of decreased numbers of the Cajal-Retzius cells marked by Reelin, with the difference from the wt being statistically significant for the homozygous *C215Y/C215Y* mice (Additional file 1: Fig. S4B, D).

**Fig. 4:**
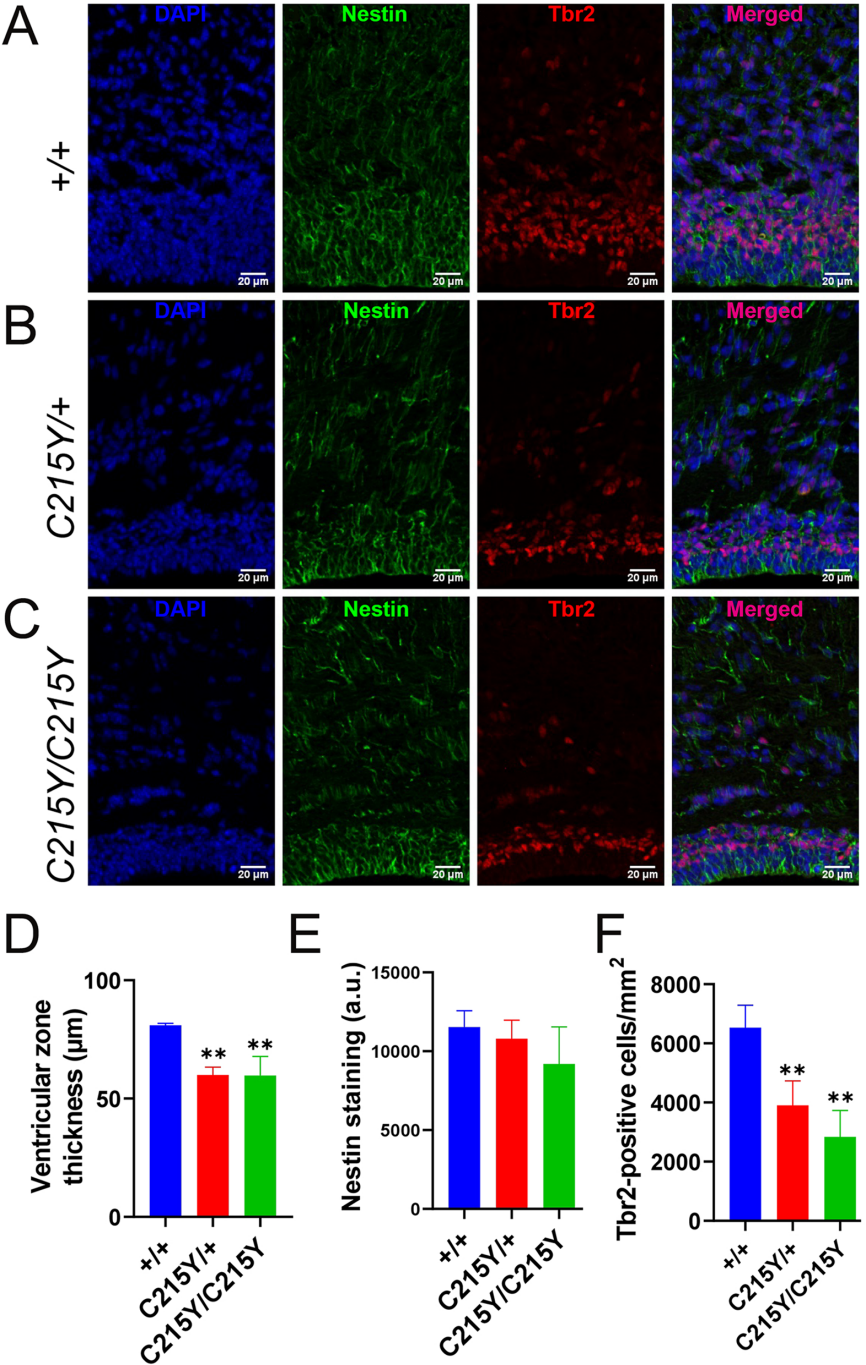
*C215Y* mutation in *GNAO1* gene affects cortical neural progenitor cells. **A**–**C** Staining of brain sections of ventricular and subventricular cortical zones from E18.5 wt (+ / + , A), *C215Y/* + (**B**), and *C215Y/C215Y* (**C**) mice for DAPI (blue), Nestin (green) or Tbr2 (red). **D** Measurement of the ventricular zone thickness. **E** Quantification of the Nestin staining intensity. **F** Quantification of Tbr2-positive cells. Data are presented as mean ± SD, n = 3–5 animals. *p < 0.05, **p < 0.01, significance from the wt is assessed by t-test. Scale bar: 20 μm

The changes we identified in the number of cells in the layers of the cortex prompted us to investigate progenitor cells for projection neurons (Fig. 4). We assessed the radial glial cells lining the lateral ventricular zone by staining for Nestin and the intermediate progenitors using the Tbr2 marker. We observed a marked decrease in the cortical thickness in lateral ventricle regions in *C215Y* mice of both homozygous and heterozygous genotypes compared to the wt littermates (Fig. 4A–D). However, intensity of the Nestin staining remained unchanged, which taken together with the overall reduction of thickness indicated a decrease in the total number of radial glial cells, rather than a change in tissue compactness (Fig. 4E). Importantly, the *C215Y/* + and *C215Y/C215Y* mice demonstrated nearly halved numbers of the Tbr2-positive cells in the ventricular and subventricular zone compared to wt littermates (Fig. 4A–C,F).

Thus, we conclude that the C215Y mutation in both heterozygous and homozygous states leads to impaired brain development mainly due to reduced numbers of the neural progenitor cells-that in turn could be an outcome of impaired differentiation or migration, but not increased apoptosis, as anti-caspase 3 staining in the cortical plate, the subventricular and the ventricular zone did not reveal any differences across the genotypes (Additional file 1: Fig. S5).

### Brain morphology and cortex structure of *G203R* E18.5 embryos reveal similarities with the *C215Y* phenotypes

Since the *GNAO1[G203R/* +*]* line could not be established due to the postnatal mortality (Fig. 1A), we processed E18.5 embryos of this genotype for IHC analysis directly after CRISPR/Cas9 transgenesis. In this manner, post-transgenesis embryos were fixed and then genotyped. Inevitably, this approach could not generate controllable and large numbers of specimen of the right genotype. In our attempt, we could generate one *G203R/* + and two *G203R/G203R* E18.5 fixed embryos. Although insufficient for statistical analysis, these experiments were deemed worthy of a qualitative “case report”, relevant for the current study as they permit comparisons with the *C215Y* animals and generalizations important to understand the disease. We thus proceeded with analysis of the *G203R* mutant animals in a manner similar we described above to *C215Y* E18.5 embryos.

H&E staining of coronal brain slices revealed strong morphological defects, such as a decrease in the brain section area in *G203R/G203R* embryos and the increase in the lateral ventricle size in *G203R/* + , the latter is similar to that in *C215Y* embryos (Additional file 1: Fig. S6A). In contrast to *C215Y*, however, the cortex thickness dropped in *G203R/* + (but not in homozygous mutants, Additional file 1: S6B). Further analysis of projection neurons in deeper layers of the cortex (Additional file 1: S6C) did not reveal changes in numbers of Ctip2-positive cells between *G203R/* + (2052 cells/mm^2^), *G203R/G203R* (2263 ± 149 cells/mm^2^, mean ± SD), as compared to the wt embryos (1842 ± 168 cells/mm^2^).

In contrast, the number of Tbr1-positive cells strongly decreased, from 2702 ± 119 cells/mm^2^ in the wt to 1640 cells/mm^2^ in the *G203R/* + and 2342 ± 323 cells/mm^2^ in *G203R/G203R*, similar to the finding for the *C215Y* genotypes. We further counted the number of Brn2-positive cells that identify the upper layers. In *G203R/* + , the number of Brn2-positive neurons (5048 cells/mm^2^) increased as compared to *G203R/G203R* (3714 ± 952 cells/mm^2^) and wt (3143 ± 287 cells/mm^2^) for II/III layers. Reelin-positive cells in the marginal zone of *G203R/G203R* (33 ± 6 cells/mm) were more numerous than in *G203R/* + (21 cells/mm) and wt (23 ± 2 cells/mm).

To assess whether these differences in the number of neurons in the cerebral cortex in *G203R* mutant mice could be due to developmental defects of neural progenitor cells, radial glial cells positive for Nestin and intermediate neuronal precursors positive for Tbr2 were evaluated, revealing a reduction in the ventricular zone thickness from 81.2 ± 2.2 µm for wt to 54.1 µm in *G203R/* + and 53.9 µm in the homozygous mutants. While we did not find any differences in the intensity of Nestin staining, the Tbr2 + precursors were decreased in the *G203R* mutants: from 6533 ± 437 cells/mm^2^ in the wt to 3600 cells/mm^2^ in *G203R/* + and 5750 ± 1626 cells/mm^2^ in *G203R/G203R*.

Comparison of the *C215Y* and *G203R* demonstrates that there is a drop in the thickness of lateral ventricle cortex and the number of Tbr1-positive and Tbr2-positive cells in both genotypes; both mutations also decrease the number of neural progenitor cells. These features highlight a common neurodevelopmental defect at the basis of the two dominant *GNAO1* mutations.

### Assessment of behavioral and motor disorders in mice with the C215Y mutation

**Fig. 5:**
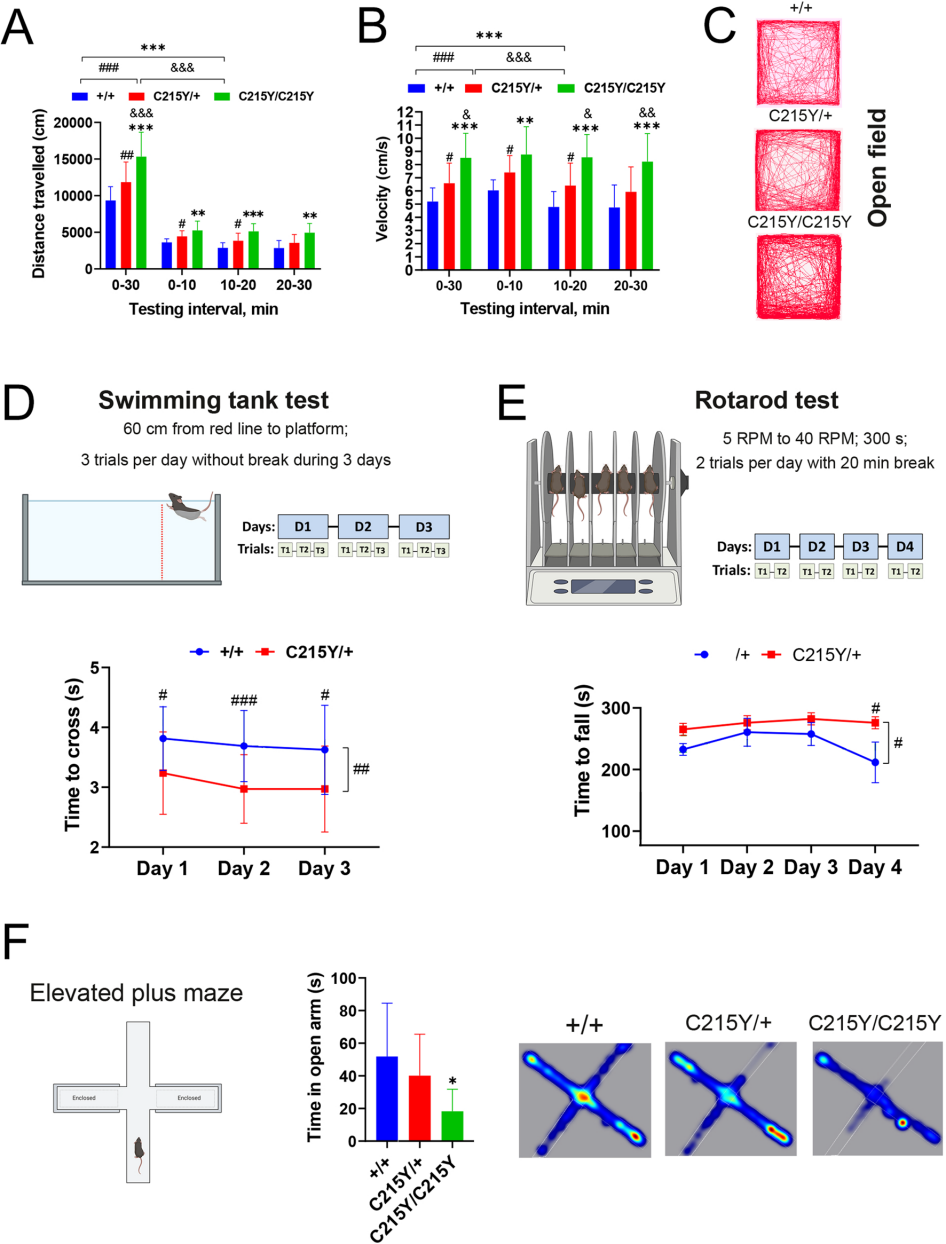
Assessment of behavioral and motor disorders in mice with the *C215Y* mutation. **A**–**C** *C215Y/* + , and even more so the homozygous *C215Y/C215Y* mice, display hyperlocomotion in the open field test. Data are shown as the distance moved by a mouse (**A**) and the velocity of the mouse (**B**) for the two mutant genotypes and their wt littermates, measured in the 0–10 min, 10–20 min, 20–30 min intervals of the experiment, and the overall 0–30 min of the experiment. Representative trail plots are shown in (**C**). **D** C215Y/ + mice outperformed the wt littermates in the swimming test. **E** In the rotarod test, *C215Y/* + mice outperformed their wt littermates. Quantification of the time to fall parameter over the four days of the experiments is shown. **F** Group mean of the time in open arms in the elevated plus maze for the three genotypes. In all panels data is given as mean ± sem. *p < 0.05, **p < 0.01, ***p < 0.001, + / + vs. *C215Y/C215Y*, #*p* < 0.05, ##*p* < 0.01, ###*p* < 0.001 + / + vs. *C215Y/* + , &*p* < 0.05, &&*p* < 0.01, &&&*p* < 0.001 *C215Y/* + vs. *C215Y/C215Y* as determined by two-way ANOVA with Dunnett’s multiple comparison test (**A**, **B**, **D**, **E**) and t-test (**F**); n = 8 (wt), 17 (*C215Y/* +) and 6 (*C215Y/C215Y*) mice

For the patients with the *C215Y/* + mutation, the reported symptoms are almost exclusively related to the motor disfunctions (Table 1) [8, 9]. We therefore chose a set of behavioral tests to characterize exploratory and motor functionalities in mice with the *C215Y* mutation. The open field was used to assay exploratory behavior and general locomotor activity in mice during 30 min. The analysis was divided in 3 stages, 10 min each, thus we separately evaluated the activity during the initial time period (0-10 min) corresponding to the exploratory activity, while the sustained locomotor activity was assessed by analysis over the entire interval of 30 min. We found that both the exploratory activity and the sustained activity were significantly increased for the heterozygous and homozygous *C215Y* mice, this effect being apparent as increase in the distance travelled and the speed of locomotion by the mutant mice (Fig. 5A–C). Homozygous mice also had significantly decreased spent time in the center of the arena compared with + / + and *C215Y/* + mice that may indicate anxiety-like behavior (Additional file 1: Fig. S7A).

Since mice of both *C215Y* genotypes showed hyperactivity in the open field test, we decided to evaluate the efficacy of risperidone as an antipsychotic drug and a drug prescribed to reduce hyperkinetic movement disorders. This drug has been shown to be effective in some patients with *GNAO1*-encephalopathy [32]. We analyzed the effects of risperidone on the behavior of the mutant animals, finding that injection of 2 mg/kg or even 0.5 mg/kg risperidone (the dosages used in the *GNAO1[R209H/* +*]* mouse study [33]) resulted in a complete immobilization of the animals for around 30 min (data not shown). The reduced dose of 0.1 mg/kg decreased the motor activity scores by several folds as compared to the vehicle-treated ones. Noteworthy, *C215Y* heterozygotes still presented statistically significant hyperactivity as compared to wt (Additional file 1: Fig. S7B; homozygotes were not tested in these experiments) – the feature also seen in the *GNAO1[R209H/* +*]* mouse study [33].

We also used the swimming tank test for monitoring the complex swimming activity. Interestingly, the *C215Y/* + mice demonstrated a significantly increased velocity in this test (Fig. 5D). Next, the accelerating rotarod test was used to assess motor coordination and balance in mice, revealing that the motor-coordination parameters of *C215Y/* + mice were more effective than those of the wt mice (Fig. 5E). The pole test aimed at  estimating the eventual bradykinesia in mice with mutations did not reveal any influence of the genotype on performance in this test (Additional file 1: Fig. S7C). Finally, the elevated plus maze test was applied to evaluate whether the *C215Y* mice presented an anxiety phenotype. We found that the homozygous mice spent less time in the open arms compared with wt (Fig. 5F), suggesting an anxiety-like phenotype which was also inferred from the open field test (Additional file 1: Fig. S7A).

### Quantitative analysis by MoSeq conforms the hyperlocomotive changes in the steady-state adult mouse behavior upon *GNAO1[C215Y]* mutations

Visually, the standard behavior of the *C215Y/* + and *C215Y/C215Y* did not differ from that of the wt littermates, unless specific behavioral tests were applied as described above. To track eventual modest locomotor dysfunctions in the routine mouse behavior, we modified the MoSeq algorithm [19, 20] as described in Materials and Methods to perform an unbiased in-depth investigation of the behavioral patterns in different genotypes. This MoSeq analysis identified 44 principal behavioral syllables that we manually assigned to interpretable patterns (e.g. Forward Move 1, Sitting 2, etc.) to simplify their grouping and representation (Additional file 1: Fig. S8). Each of the syllables was characterized by three principal characteristics: (i) frequency (min^−1^) as the measure of how often the syllable is initiated by the animal per minute of activity; (ii) mean length (sec) as the average duration of an uninterrupted syllable; (iii) proportion (%) as the overall percentage of time the animal used a given syllable over the entire period of recording.

We performed a robust statistical analysis of these characteristics of syllables between females of *C215Y/* + , *C215Y/C215Y*, and the littermate wt genotypes (Additional file 1: Figs. S8 and S9). The most notable observation is a significant decrease in the *C215Y* mutants of the proportion of syllables #26 and #32 (Sitting 8 and Sitting 10), being the main sitting postures of the animals. This decrease correlates with the allele number. For these syllables, in *C215Y/C215Y* mice we also observed the corresponding increase in their frequency, and for the syllable 26—an additional drop in its mean length (Additional file 1: Fig. S9). This indicates that the mutation reduces the time the mice are motionless, which is additionally corroborated by the respective growth of the frequency and the proportion of the syllable #28 (Forward move 8) designating nearly half of the forward moves of the animals, and of the syllable #1 (Rearing stand 1). Interestingly, while the homozygous animals perform syllable #1 (Rearing stand 1) more frequently and more often, the heterozygous ones demonstrate a significantly increased length of the stand.

Additional information could be obtained from the network analysis, where the nodes represent syllables and edges—transitions between them. On the Additional file 1: S10A-C, the size of the node and its label represent the syllable proportion as shown in the Additional file 1: Fig. S8, while the edge thickness corresponds to the proportion of a given transition relatively to the entire number of syllable transitions in the animals’ behavior. We observed that syllables #26 and #32 (Sitting 8 and Sitting 10) are frequently interchanged in wt mice with nearly 2% of all pattern transitions occurring only between these two. However, in the *C215Y/* + and *C215Y/C215Y* animals this proportion is reduced and the animals are more frequently transiting into other syllables, e.g. related to forward movements or rearing, which is reflected by the growth in transitions between syllables #27 and 28 (Forward moves 7 and 8), or #32 and #7 (Sitting 10 to Forward move 7). Interestingly, while both syllables #26 and #32 (Sitting 8 and Sitting 10) are decreased in the mutants, the proportion of only one type of the forward move is affected, indicating that this hyperlocomotive behavior is specific for a particular type of movement (Additional file 1: S10B, C).

In summary, this deep behavioral analysis makes it clear that the *C215Y* mutant animals exert the hyperlocomotive behavior also under the unchallenged conditions by reducing the movement patterns associated with sitting and increasing those associated with forward moves. The number and significance of the behavioral changes observed correlate with the allele number: *C215Y*/ + animals score overall 7 significant differences in syllable characteristics as compared to the wt, with this number growing to 23 for the *C215Y/C215Y* animals. Altogether, this in-depth analysis of the regular mouse behavior corroborates the findings of the dedicated behavioral tests: *C215Y* mutant mice are hyperactive and hyperlocomotive. This phenotype can be viewed as the mouse analog of the excessive and uncontrolled motor activity of human *GNAO1[C215Y/* +*]* patients (Table 1) [8, 9].

### Lack of epileptic manifestations in *C215Y/* + animals

Patients carrying the *C215Y GNAO1* mutation do not reveal signs of epilepsy [8, 9] (Table 1), unlike patients with several other point mutations in *GNAO1*. In agreement with these clinical findings, we did not observe any signs of behavioral seizures (such as facial automatisms, myoclonus, jumping and running or tail or hindlimb extension) in *C215Y/* + or *C215Y/C215Y* mice during the animal handling and behavioral assessment procedures. To corroborate on these phenotypic observations, electroencephalography (EEG) recordings were performed on wt and *C215Y/* + mice (see Materials and Methods), that did not reveal any epilepsy-related activity in either genotype (Additional file 1: Fig. S11A-C). Careful visual inspection of the EEG sessions did not reveal any epileptiform episodes such as seizure-like or other types of pathological patterns reminiscent of paroxysmal discharges in controls and in the mutant mice. We did not observe alterations in the EEG activities as compared to the control wt mice. Measuring the number of fast-ripples (FRs) at all surface electrodes in 3 controls and 3 mutant mice, FRs were only detected at electrodes above the somatosensory regions in both genotypes and at a similar range (median and IQ range: Control: 0.16, 0.11, 0.3; Mutant: 0.2, 0.03, 0.23) suggesting that only physiological FRs were detected.

To further investigate the epileptic potential of the *C215Y/* + mice, we performed the pentylenetetrazole (PTZ)-induced kindling experiment (Additional file 1: Fig. S11D). As Additional file 1: Fig. S11E reveals, at two increasing doses of PTZ, seizures were progressively induced, but the seizure score was indistinguishable between the *C215Y/* + mice and their wt littermates.

## Discussion

First reported in 2013, dominant mutations in *GNAO1* cause a spectrum of early-onset neurological deficiencies in affected children, encompassing, depending on the exact mutation, severe motor disabilities, epileptic seizures, developmental delay, intellectual disability, and progressive brain atrophy [1–4]. The patients often require intensive care after birth, as well as gastrostomy due to difficulties in feeding [1, 3]. As of today, no curative therapy exists for *GNAO1* encephalopathy patients, with symptomatic treatments at best demonstrating partial and temporary effects [3, 10, 11].

Proper animal models are instrumental to decipher disease mechanisms and to develop eventual treatment regimens. *GNAO1* encephalopathy patients harbor one wt and one mutant allele of *GNAO1*; as the majority of the clinical cases described do not reveal mosaicism [2, 4, 7, 11, 34], the de novo mutations in *GNAO1* supposedly arise in the gamete of one of the parents. Thus, the resulting dominant action of the pathogenic point mutation affects the patient development-and primarily the patient’s CNS development—through all stages of embryonic and postnatal development. Further, equal (or at least similar) levels of expression of the mutant and wt *GNAO1* variants are observed (see Additional file 1: Fig. S1 or [33]). These considerations imply that mere over- or mis-expression of a mutant *GNAO1*, either ubiquitous or restricted to a particular brain area or a particular developmental time window, cannot represent a model adequately recapitulating the human patients. Such an adequate animal model of *GNAO1* encephalopathy can only be generated through introduction (e.g. with CRISPR/Cas9) of the exact pathogenic point mutation into one allele of the endogenous *GNAO1* locus, keeping the other allele and the expression control mechanisms intact, and observing the resultant dominant phenotypes. Such an approach has so far been proven successful in two studies in mice: an *GNAO1[R209H]/* + mouse model [33] and the two mouse models described in the current manuscript: *GNAO1[G203R]/* + and *GNAO1[C215Y]/* + . Further, similar modeling has proven successful in *Drosophila* (*Gαo[G203R]/* + model with reduced locomotion and life span, responding to a pharmacological rescue [35]). The modeling of *GNAO1* encephalopathy has also been performed in *C.elegans*, with the nematodes heterozygous for S47G or A221D mutations revealing the dominant "unlaid eggs" phenotype indicative of effects in motor neurons, while the heterozygous G42R or R209C mutations produced the dominant aldicarb hypersensitivity effects [36, 37]. In contrast to these knock-in modeling, over/mis-expression of mutant Gαo on top of the two wt alleles can be envisioned. For example, overexpression of Gαo[G203R] or Gαo[R209C] in striatal neurons has resulted in impaired locomotor behavior in mice [38]. This simplified approach may serve as a tool to provide insights on the functioning of the G protein and its mutants [38], but its relevance as the disease model is unclear. It is interesting to note in this regard that the hyperactivity we observe in the *C215Y/* + mice is not associated with anatomical defects in the striatum (Additional file 1: Fig. S2) but in the motor cortex (Fig. 2).

The two mouse models created and analyzed in our current study represent human patients at the two extremes of the *GNAO1* encephalopathy clinical manifestation spectrum. On the one hand, G203R is one of the hotspot mutations in the disease that leads to the strongest and earliest in the onset deficiencies combining both motor dysfunctions and epileptic seizures, further accompanied by developmental delay and intellectual disability. On the other side of the spectrum, C215Y mutations lead to neither epilepsy nor developmental/intellectual delays, but present clinically with motor abnormalities that manifest much later in life. These differences in the clinical manifestations of the two mutations are fairly reflected in the mouse models already at the level of the survival. Indeed, *G203R/* + mice are neonatally lethal with the longest surviving pups reaching the age of 14 and 4 days. Although this severe neonatal lethality did not permit assessment of eventual epileptic seizures, it is worth mentioning that another mouse model—*GNAO1[G184S]/* + harboring an activating mutation not seen in human patients–displayed spontaneous lethality preceded by seizures [39]. In contrast, *C215Y/* + mice are viable and fertile. Not revealing any epileptic phenotypes, the *C215Y/* + mice proved hyperactive and hyperlocomotive in a broad panel of behavioral tests, similarly to the previously described *R209H/* + mouse model [33]; the homozygous loss-of-function *GNAO1*^*−/−*^ mice also have been reported to display hyperactivity [27]. Uniquely, we could observe this hyperactivity not only in the standard tests like the open field or swimming tests, but also upon analysis of the unchallenged mouse behavior, assessed in-depth through a dedicated MoSeq video monitoring and analysis algorithm we customized explicitly for this study. This hyperlocomotion, seizure-free phenotype can be viewed as the mouse analog of the excessive and uncontrolled motor activity of the human *C215Y* patients [8, 9], similarly to the R209H mutation in patients and mice that leads to motor dysfunction but not epilepsy [33].

Regional brain malformations and atrophy, sometimes progressive, have been described in *GNAO1* patients [5, 6, 11, 40–42]. It has so far been impossible, however, to decode whether these malformations are the cause or the consequence of brain malfunctioning such as epileptic onsets. In a broader sense, it has been unknown if (and to which extent) *GNAO1* encephalopathy is a neurodevelopmental disorder. In our study, we provide the first insights into this key aspect of the disease etiology. Remarkably, in both the *G203R* and *C215Y* mouse models, despite the differences we observed among them, significant prenatal brain development defects could be identified. These defects manifested in strong enlargement of lateral ventricles and reduced numbers of Tbr1-positive and Tbr2-positive cells. The decrease in the neural progenitor cells could result from impaired differentiation or migration (but not increased apoptosis, see Additional file 1: Fig. S5). Interestingly, we recently described a *GNAO1[Q52R]* patient with periventricular nodular heterotopia seen on MRI, that represented a focal neuronal migration defect [11]. The commonality of neurodevelopmental abnormalities in the two mutants, despite their distinct behavioral and clinical manifestations, highlights a common neurodevelopmental defect at the basis of the two dominant *GNAO1* mutations and potentially of *GNAO1* encephalopathies in general. The fact that at later stages the two mutants deviate from each other into a mild encephalopathy (C215Y) *vs*. a severe one (G203R) both in patients and in mice may suggest that the Gαo-controlled biochemical / physiological processes continue to be affected postnatally, differently for the two mutations. It might also be hypothesized that the neurodevelopmental abnormalities in the motor cortex, common for the two *GNAO1* mutations, represent the ‘basic’ misfunction in *GNAO1* encephalopathy and underlie the movement defects in the patients. Further aberrations ‘added’ later in development in the carriers of more severe mutations such as G203R then mediate additional defects like epilepsy.

Our work thus provides crucial insights into the etiology of this rare yet devastating disease, identifying it as to a large extent neurodevelopmental disorder. This understanding and the animal models we established shed light on the normal and pathological roles of Gαo in the nervous system and will pave the way for eventual therapeutic developments.

## Supplementary Information

**Additional file 1**: **Fig. S1**. Transcription of G203R and C215Y alleles. (A) Schematic exon-intron structures of *GNAO1*. Black and white boxes mark the coding and noncoding parts of the exons respectively. The drawing of the exons is presented in scale. Digits inside the exons indicate their precise length in base pairs. Red arrows flag mutations in the 6^th^ exon. Primers used in RT-PCR are designated with black horizontal arrows. (B) Agarose gels with RT-PCR products obtained for different genotypes with the primers 1 and 2 as shown on panel (A). As expected, all resultant products migrate in gels as 160bp bands. (C) Sequences of RT-PCR products for *GNAO1* mutants and the wt (+/+) control. Codons, amino acids with their numbers are shown above the sequence of the wt. Black dashed vertical line demarcates the 5^th^ and 6^th^ exons. Red dashed vertical lines designate codons for the 203^rd^ and 215^th^ amino acids of Gαo. Note that the peak height of the mutant nucleotide is nearly identical to that of wild-type in both mutants, speaking of near-equal expression of both alleles. **Fig. S2**. Normal striatum morphology in ***C215Y*** mutants. (A) Staining of brain sections with striatum identified with the Ctip2 marker (marks GABAergic medium-sized spiny neurons) from wt (+/+), *C215Y/+*, and *C215Y/C215Y* E18.5 embryos. (B) DAPI staining of the same sections. Note the enlarged lateral ventricles (right to the striatum) in the *C215Y* mutants. (C) Quantification of Ctip2-positive cells. (D) Quantification of the cell density by DAPI-stained cells in striatum. Data is presented as mean ± SD; n = 3 animals. Scale bar: 0.5mm. **Fig. S3**. Normal hippocampus morphology in ***C215Y*** mutants. (A) Cresyl violet staining of coronal brain sections from wt (+/+), *C215Y/+*, and *C215Y/C215Y* E18.5 embryos does not reveal significant changes in the morphology of hippocampus. Scale bar: 100μm. (B) Magnified photomicrographs of CA1 subfield of the hippocampus, marked with a rectangle in (A). Scale bar: 20μm. (C) The graph summarizing CA1 neuronal counts as Data is presented as mean ± SD; n = 3 animals. **Fig. S4**. Analysis of the upper layer cortical neurons and Cajal-Retzius cells in ***C215Y*** mutants. Staining of motor cortex brain sections from E18.5 wt (+/+), *C215Y/+*, and *C215Y/C215Y* mice for DAPI and Brn2 (layers II-V (A)), or DAPI and Reelin (marker of Cajal-Retzius cells, (B)). (C) Quantification of Brn2-positive cells. (D) Quantification of Reelin-positive cells. Data is presented as mean ± SD; n= 3-5 animals; **p<0.01, significance from the wt is assessed by t-test. Scale bar: 20μm (A) and 50μm (B). **Fig. S5**. Normal levels of apoptosis in C215Y brain sections. The distribution of cleaved caspase-3 (CC3)-positive cell in the brain sections from E18.5 wt (+/+), *C215Y/+*, and *C215Y/C215Y* mouse embryos. (A) Representative immunohistochemistry pictures of cleaved caspase-3 (CC3)-positive cells (marked with arrows) in the cortical plate, scale bar 20μm. The counting of caspase-3 positive cell in the cortical plate (B), the subventricular zone (C) and the ventricular zone (D). Data is presented as mean ± SD, n= 3; t-test did not reveal any statistically significant differences from wt. **Fig. S6**. ***G203R*** mutation affects brain morphology, neurons in deep and upper cortical layers as well as neural progenitor cells. (A) Hematoxylin and eosin staining of coronal brain sections from *G203R/+*, *G203R/G203R* and wt (+/+) littermates at embryonic day 18.5 shows enlargement of lateral ventricles in *G203R/+* and reduction in *G203R/G203R* mice. (B) Coronal sections through the motor cortex show differences in cortical thickness between wt and *G203R/+* mice. (C) Staining of brain sections (at motor cortex) from E18.5 *G203R/+*, *G203R/G203R* and +/+ littermates for Tbr1 (layer VI), Ctip2 (weak in layer VI, strong in layer V), Brn2 (layer II/III), Reelin (marker of Cajal-Retzius cells), Tbr2 (marker of intermediate neuronal progenitor cells) and Nestin (marker of radial glial cells). Scale bars: 1mm (A), 100µm (B, C). **Fig. S7**. Additional data for the open field and pole tests. (A) Open field test: group mean of the time in the center of open field arena for the +/+, *C215Y/+*, and *C215Y/C215Y* mice. (B) *C215Y/+* mice remained more mobile than wt in the open field test despite the overall suppression of motility induced by risperidone treatment (0.1mg/kg). Data is shown as distance moved by a mouse in the period of 30min of the experiment. (C) Pole test: means of time to a T-turn and time to descend in the pole test for the three genotypes. Data are presented as in Figure 5. **Fig. S8**. MoSeq quantification of the exploratory behavior under unchallenged conditions. The modified MoSeq algorithm was applied to monitor and analyze behavior of *C215Y/+* and *C215Y/C215Y* female mice, alongside with their wt littermates, under non-challenged conditions in a square open field. The heatmaps demonstrate comparisons among the three genotypes for the syllable (behAdditional file 1: avioral pattern) frequency (number of times this syllable starts per minute of activity), mean length (the time the syllable lasts before changing to another syllable) and its overall proportion in percent over the entire analyzed time. Statistical significance was assessed by Dunn's multiple comparison test for each syllable and is shown as a number of compared group on top of the heatmap cell in case of a significant difference (*p* value <0.05), numbers are shown side by side in case of significant differences in 2 groups. Syllable description is provided below the heatmaps. **Fig. S9**. Detailed view of selected syllables representing majority of forward, sitting and rearing-related movements. The main characteristics of these syllables shown in **Fig. S9** as a heatmap represented here as bar charts. Significance was assessed as in **Fig. S7**, *p<0.05 , **p<0.01, ***p<0.001. **Fig. S10**. Networks analysis of MoSeq behavioral data. Transitions between behavioral syllables for wt (A), *C215Y/+* (B), and *C215Y/C215Y* (C) animals. The syllables are shown as nodes and grouped by their gross appearance. The node size corresponds to the overall proportion of the syllable over the recording time (as shown on heatmaps in **Fig. S8**), nodes of syllables showing significant differences in at least one aspect (frequency, length or proportion,) are marked in red. Edge thickness corresponds to a proportion (in %) of a given transition pair among all recorded transitions for the genotype, purple numbers are shown for edges with thickness > 0.2. Edges with thickness <0.01 represent rare transitions and are thus not shown. **Fig. S11**. Absence of epileptic manifestations in ***C215Y/+*** mice. Surface EEG recordings in control and mutant awake mice. (A) The head-fix epicranial EEG recording setup. The right image illustrates the localizations of the electrodes over the mouse brain. In red, the electrodes positioned above the somatosensory cortex regions. (B) Examples of 10sec windows of background EEG recorded in both control and mutant mice. (C) Illustrative examples of physiologic fast ripples (FRs) recorded over the somatosensory cortex in control and mutant mice as visible in the raw signals and in the signal filtered between 200-550Hz. (D, E) Pentylenetetrazole (PTZ)-induced mouse kindling reveals no difference between *C215Y/+* and wt mice. Scheme of the experiment is shown in (D). In (E), seizure score quantification at three different injections of PTZ. Data are shown as mean ± SD, n=5 (wt) and 7 (*C215Y/+*) mice.
