# Supplementary material for: Mouse models characterize GNAO1 encephalopathy as a neurodevelopmental disorder leading to motor anomalies: from a severe G203R to a milder C215Y mutation
Source: Acta Neuropathol Commun. 2022 Jan 28;10:9. doi: 10.1186/s40478-022-01312-z (PMC8796625; doi:10.1186/s40478-022-01312-z)
Supplement: Supplementary file 1 — Additional file 1: Fig. S1. Transcription of G203R and C215Y alleles. (A) Schematic exon-intron structures of GNAO1. Black and white boxes mark the coding and noncoding parts of the exons respectively. The drawing of the exons is presented in scale. Digits inside the exons indicate their precise length in base pairs. Red arrows flag mutations in the 6th exon. Primers used in RT-PCR are designated with black horizontal arrows. (B) Agarose gels with RT-PCR products obtained for different genotypes with the primers 1 and 2 as shown on panel (A). As expected, all resultant products migrate in gels as 160bp bands. (C) Sequences of RT-PCR products for GNAO1 mutants and the wt (+/+) control. Codons, amino acids with their numbers are shown above the sequence of the wt. Black dashed vertical line demarcates the 5th and 6th exons. Red dashed vertical lines designate codons for the 203rd and 215th amino acids of Gαo. Note that the peak height of the mutant nucleotide is nearly identical to that of wild-type in both mutants, speaking of near-equal expression of both alleles. Fig. S2. Normal striatum morphology in C215Y mutants. (A) Staining of brain sections with striatum identified with the Ctip2 marker (marks GABAergic medium-sized spiny neurons) from wt (+/+), C215Y/+, and C215Y/C215Y E18.5 embryos. (B) DAPI staining of the same sections. Note the enlarged lateral ventricles (right to the striatum) in the C215Y mutants. (C) Quantification of Ctip2-positive cells. (D) Quantification of the cell density by DAPI-stained cells in striatum. Data is presented as mean ± SD; n = 3 animals. Scale bar: 0.5mm. Fig. S3. Normal hippocampus morphology in C215Y mutants. (A) Cresyl violet staining of coronal brain sections from wt (+/+), C215Y/+, and C215Y/C215Y E18.5 embryos does not reveal significant changes in the morphology of hippocampus. Scale bar: 100μm. (B) Magnified photomicrographs of CA1 subfield of the hippocampus, marked with a rectangle in (A). Scale bar: 20μm. (C) The graph [file 40478_2022_1312_MOESM1_ESM.pdf]

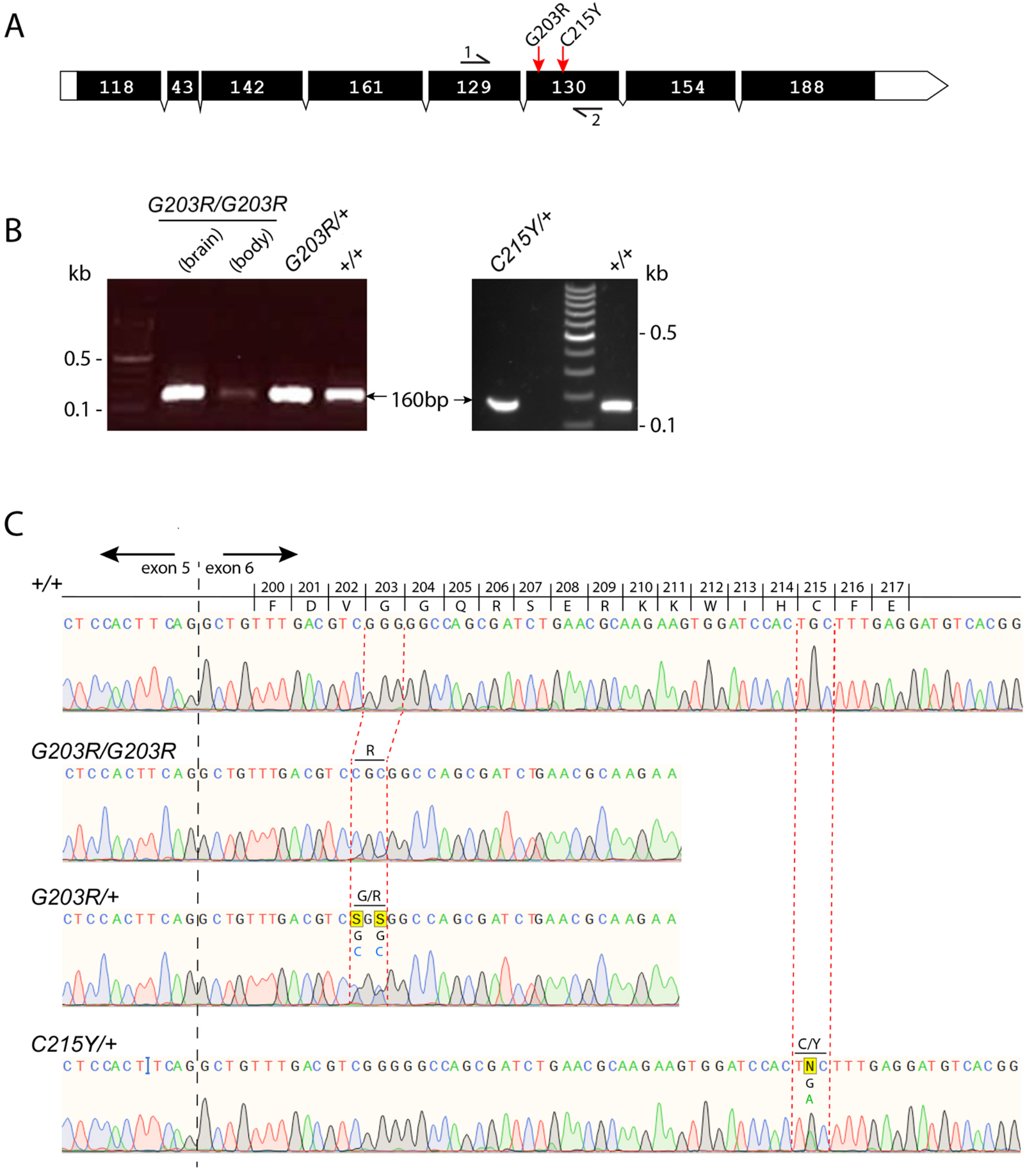

Silachev *et al.*, Supplementary Fig. S1

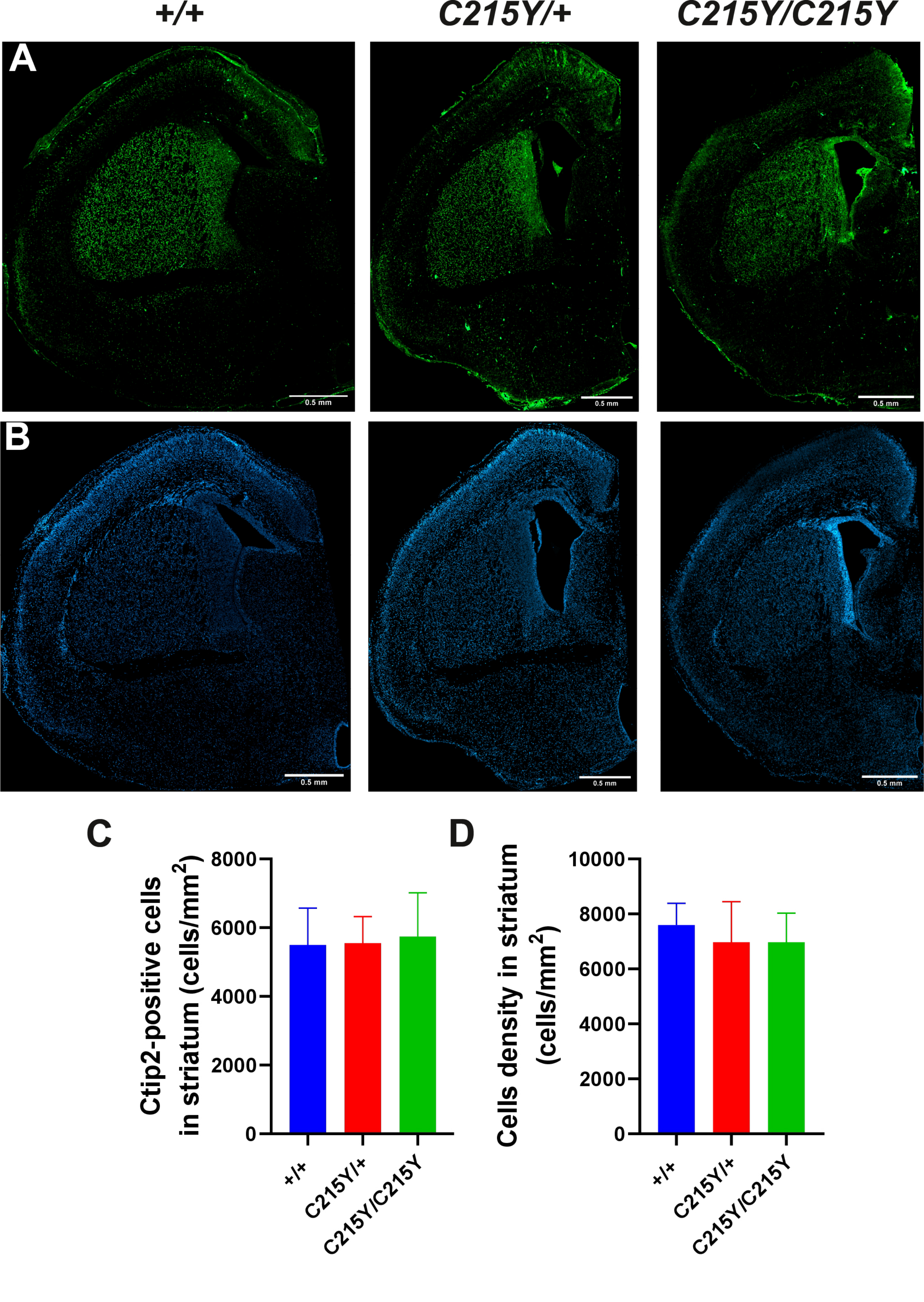

Silachev *et al.*, Supplementary Fig. S2

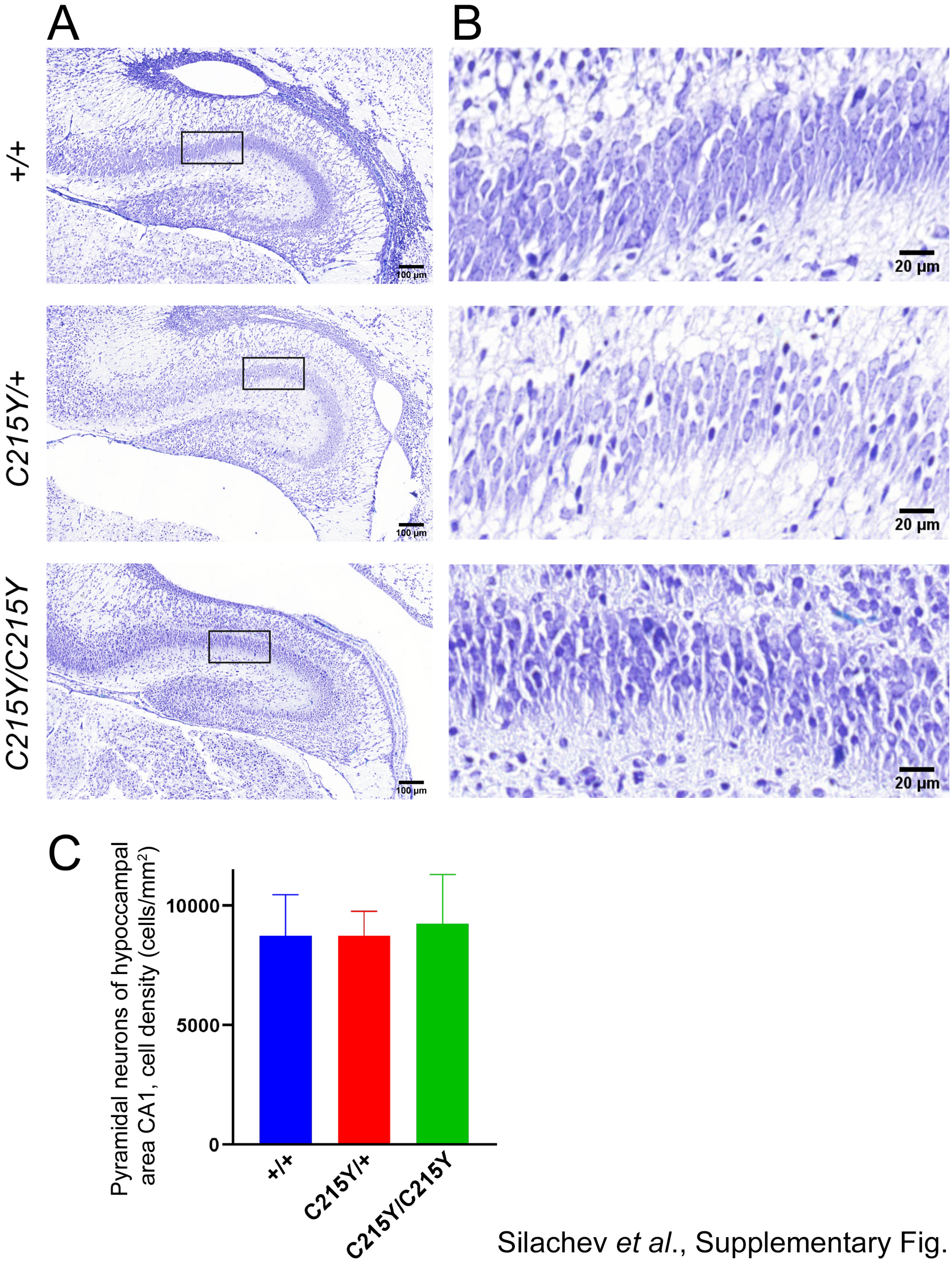

Silachev *et al.*, Supplementary Fig. S3

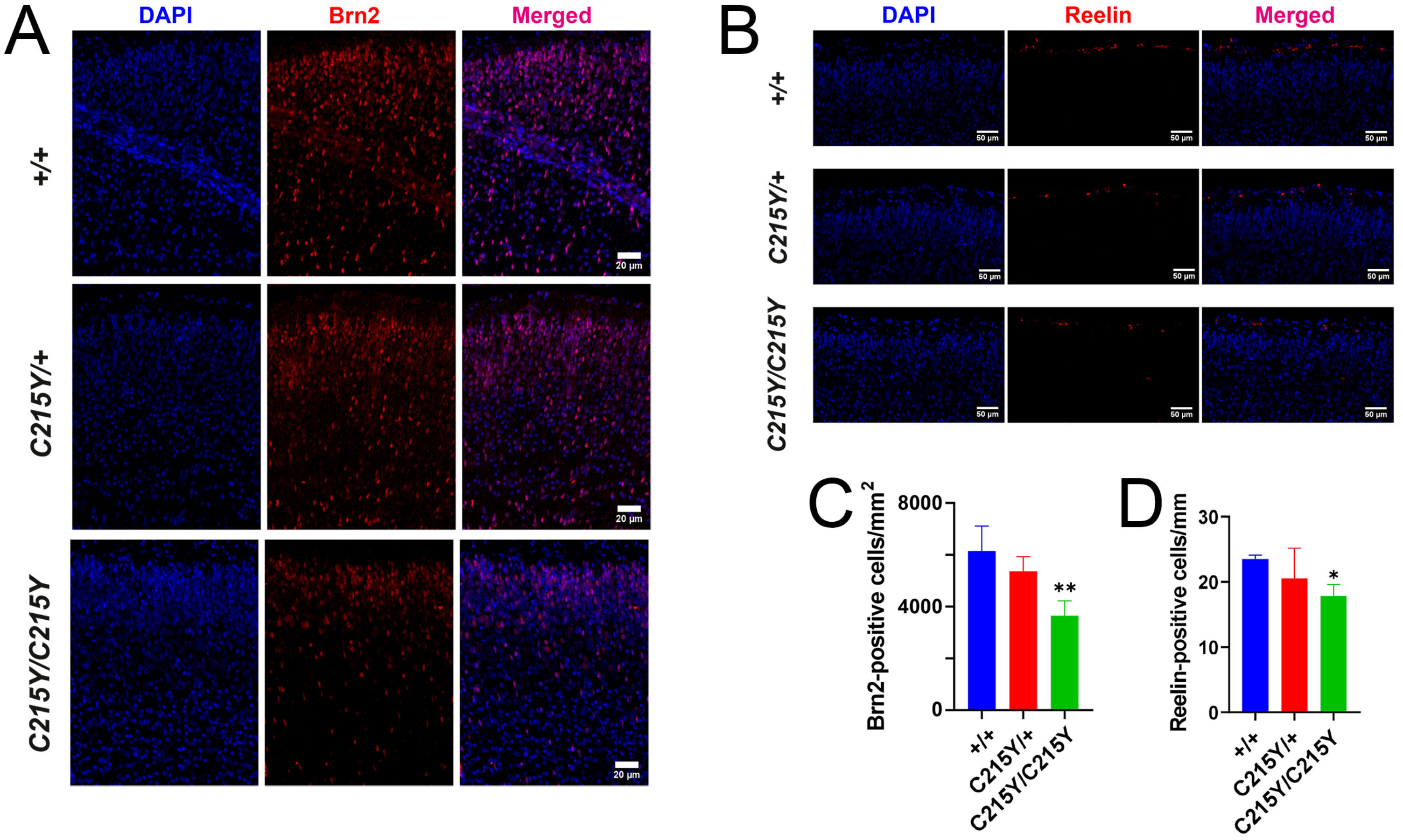

Silachev *et al.*, Supplementary Fig. S4

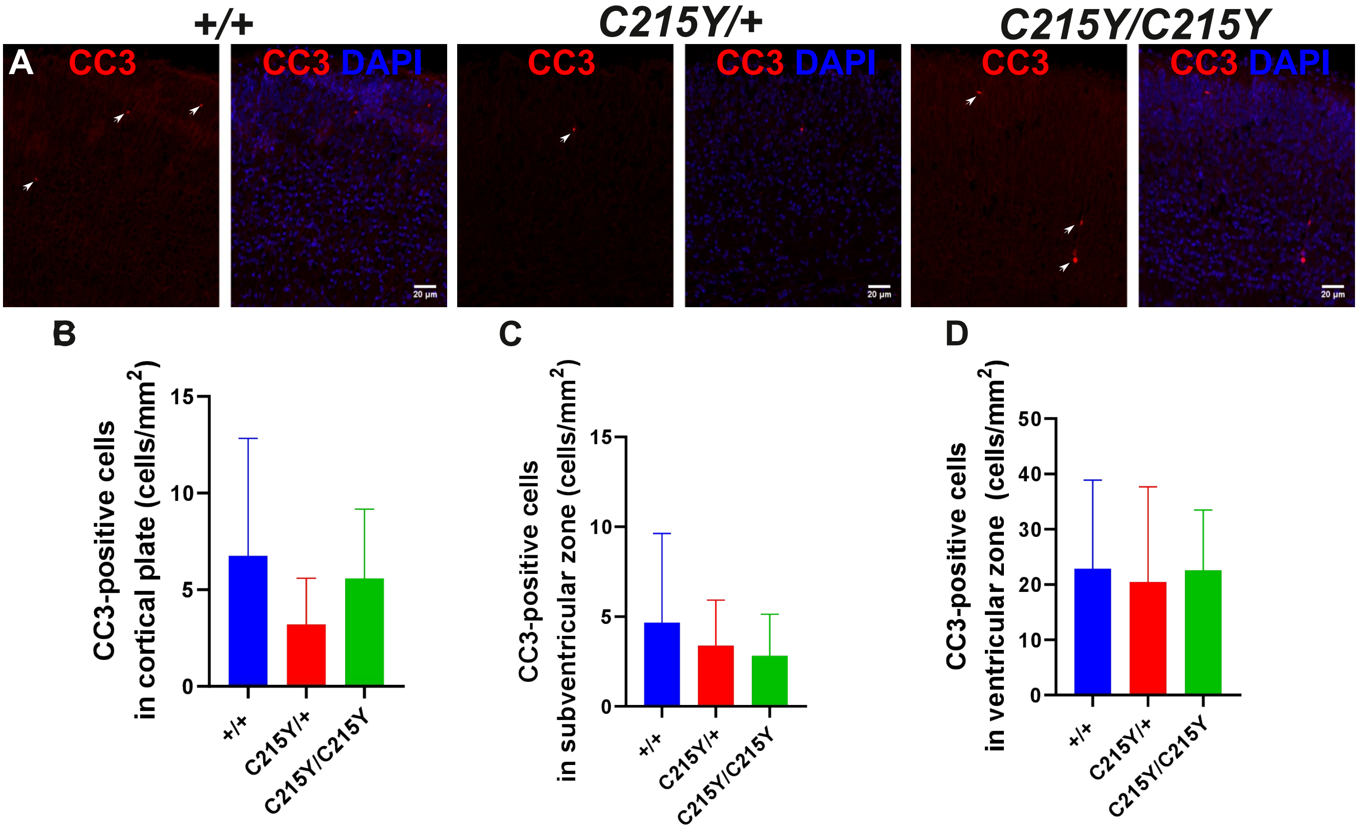

Silachev *et al.*, Supplementary Fig. S5

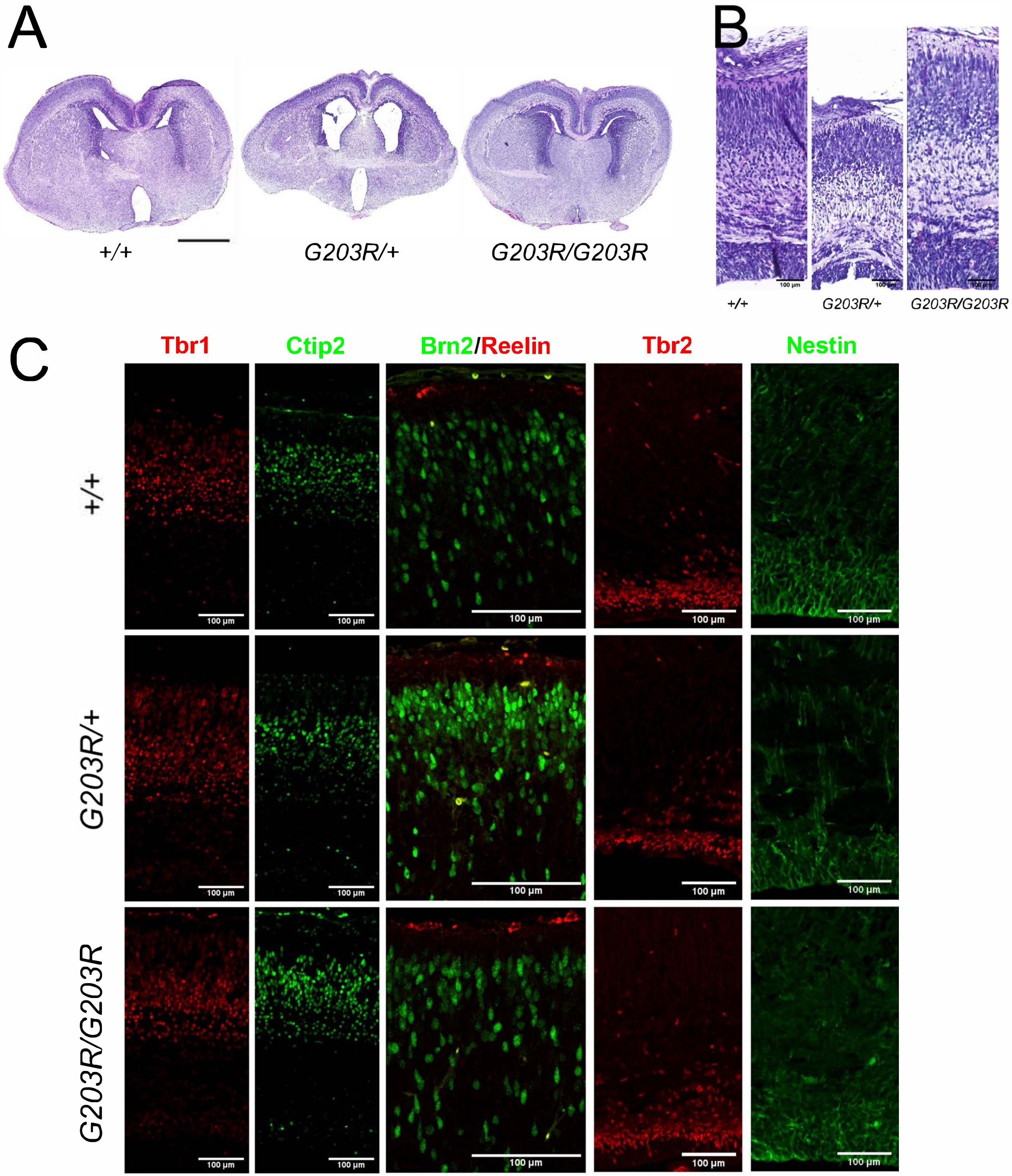

Silachev *et al*., Supplementary Fig. S6

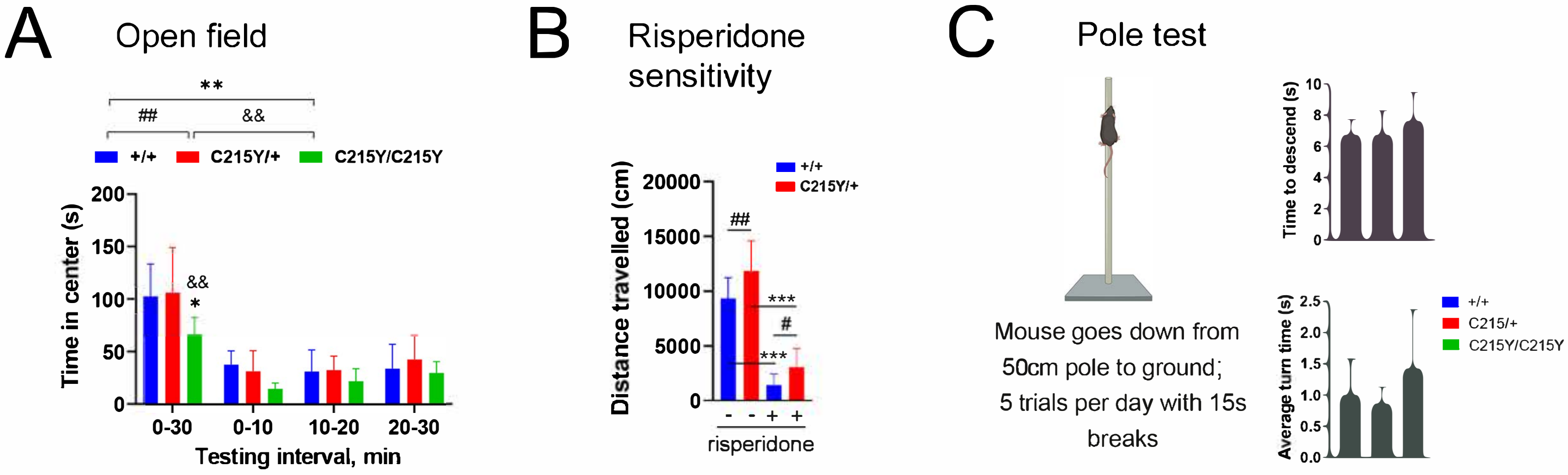

Silachev *et al.*, Supplementary Fig. S7

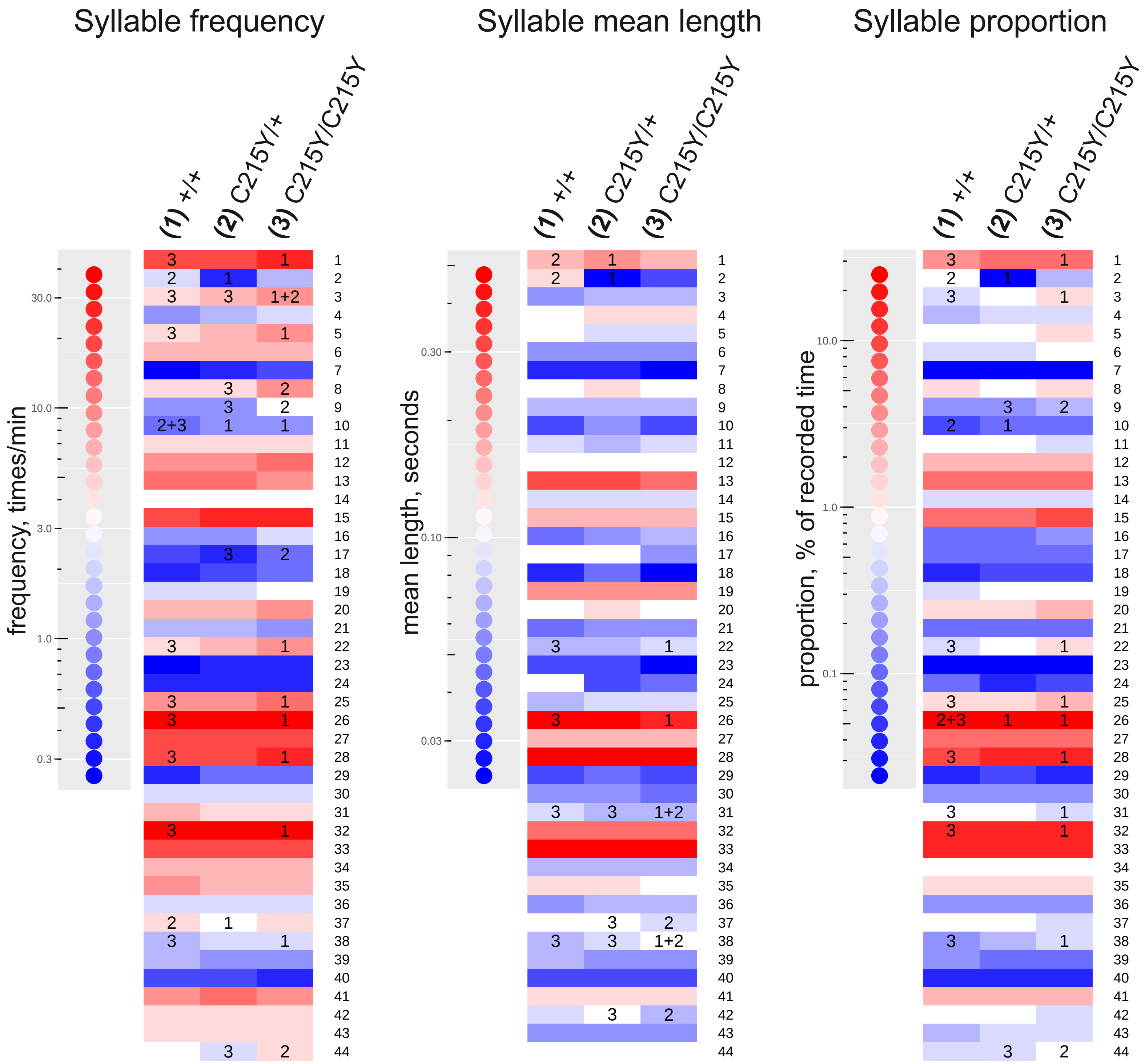

Syllable description by gross appearance:

1	Rearing – stand 1	12	Rearing – final 2	23	Forward move 6	34	Sitting 11
2	Transient 1	13	Sitting 2	24	Sitting 6	35	Pause 3
3	Rearing move 1	14	Forward move 3	25	Sitting 7	36	Transient 4
4	Rearing – stand 2	15	Rearing – stand 4	26	Sitting 8	37	Forward move 11
5	Rearing -stand 3	16	Forward move 4	27	Forward move 7	38	Transient 5
6	Rearing – raising	17	Sitting 3	28	Forward move 8	39	Forward move 12
7	Sitting 1	18	Transient 2	29	Transient 3	40	Sitting 12
8	Rearing – final 1	19	Sitting 4	30	Forward move 9	41	Sitting 13
9	Rearing – start	20	Sitting 5	31	Sitting 9	42	Transient 6
10	Forward move 1	21	Forward move 5	32	Sitting 10	43	Forward move 13
11	Forward move 2	22	Pause 1	33	Forward move 10	44	Forward move 14

Silachev et al., Supplementary Fig. S8

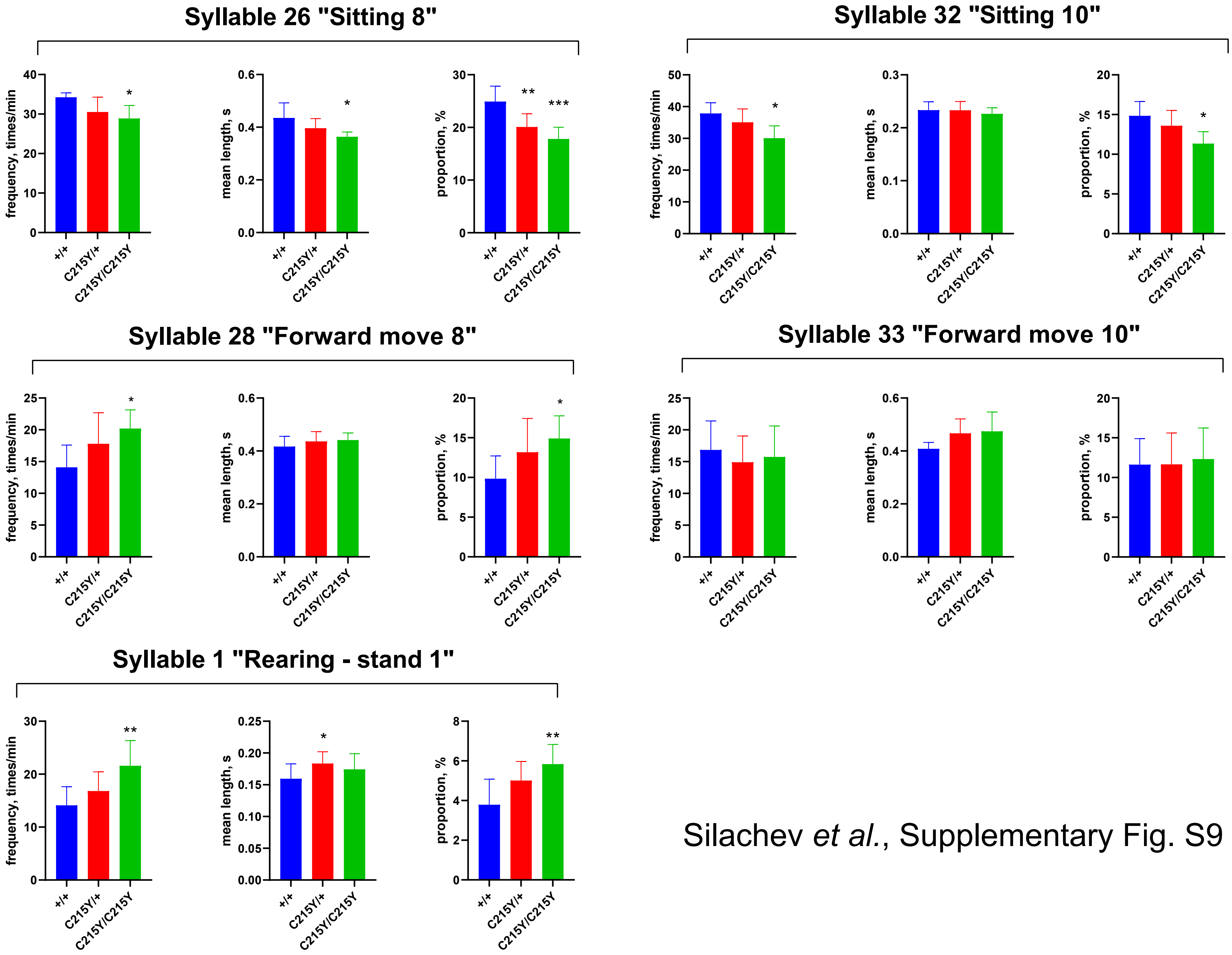

Silachev *et al.*, Supplementary Fig. S9

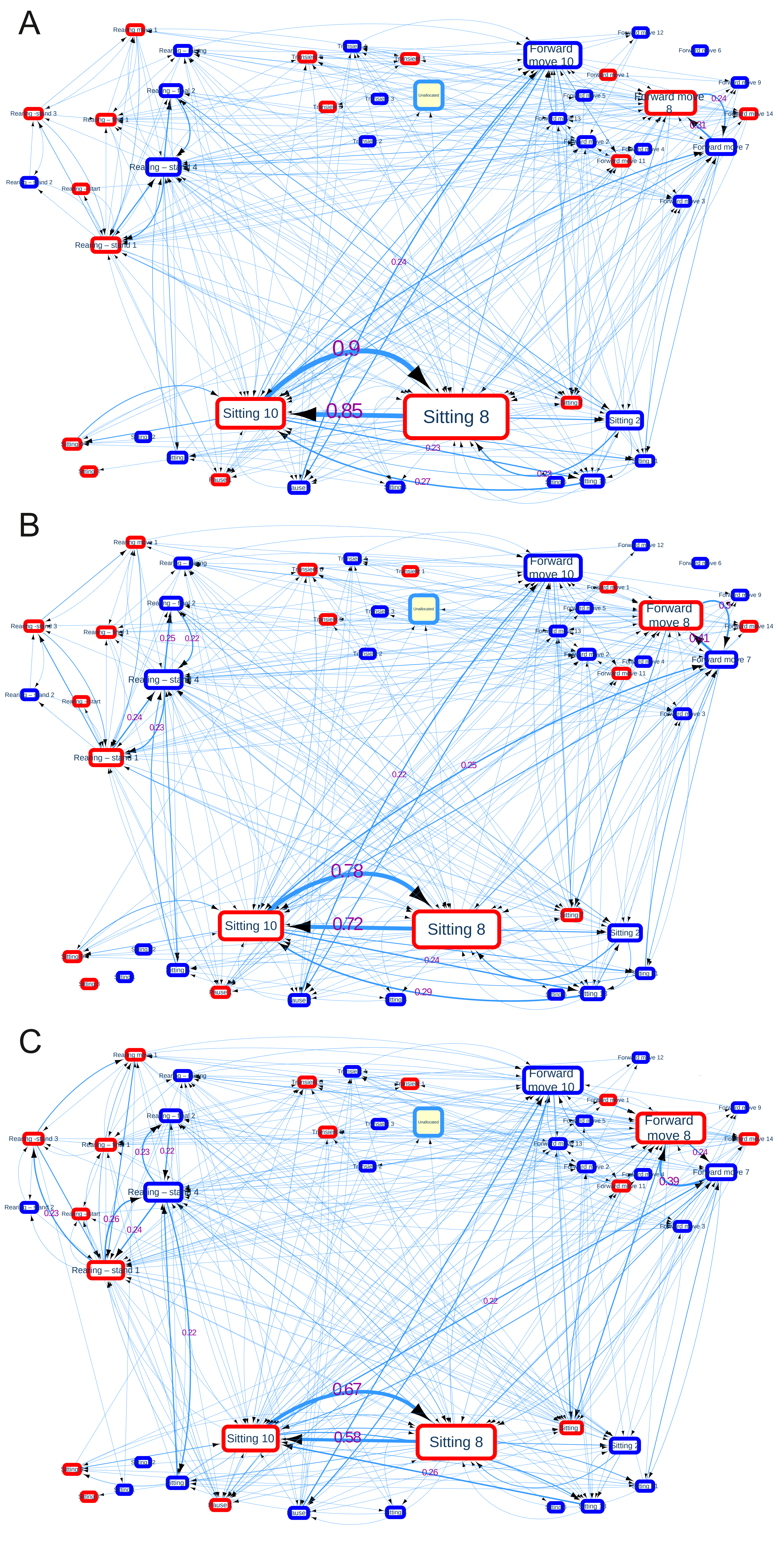

Silachev *et al*., Supplementary Fig. S10

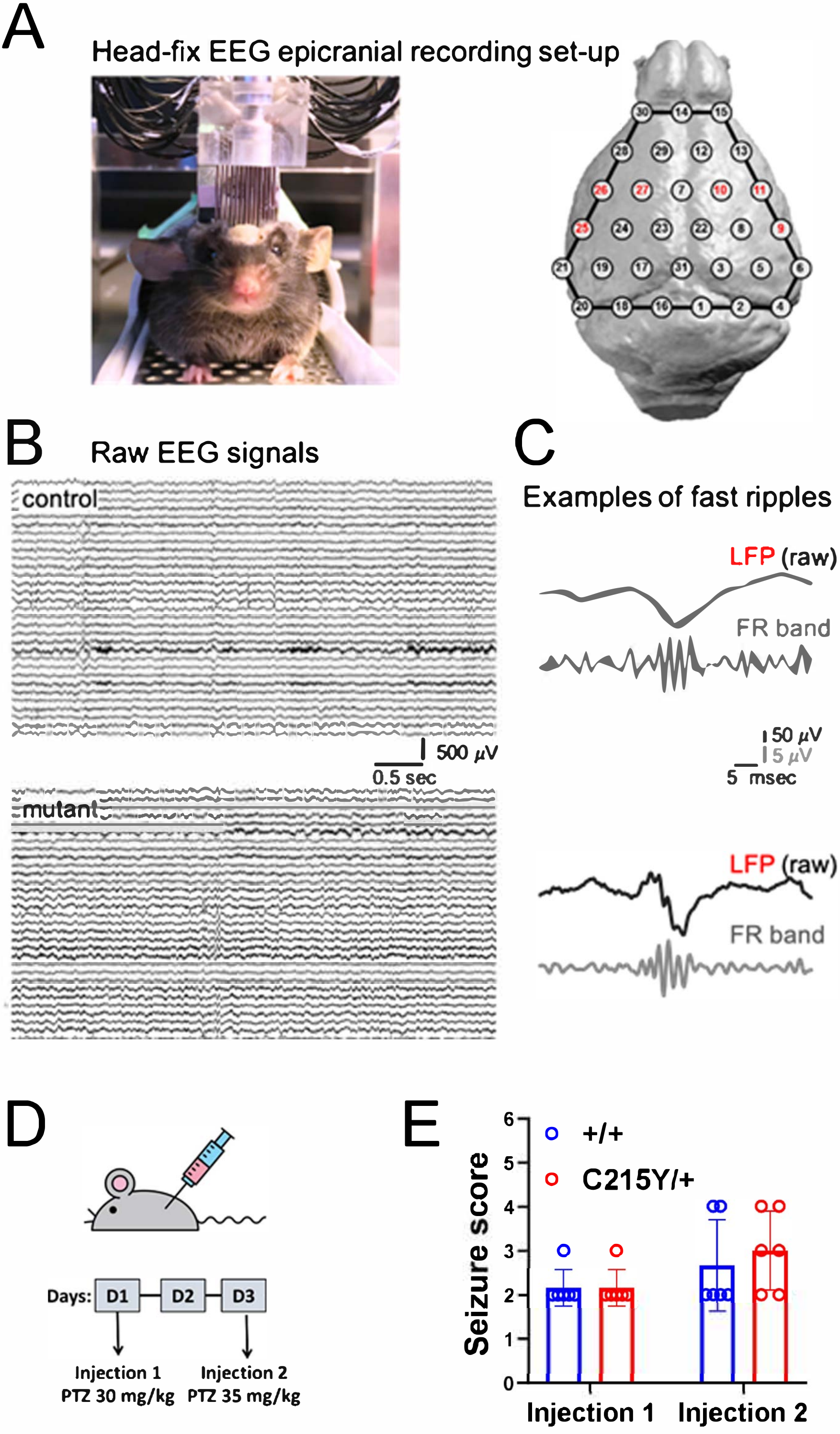

Silachev *et al.*, Supplementary Fig. S11
